# Morphology of the maxilla informs about the type of predation strategy in the evolution of Abelisauridae (Dinosauria: Theropoda)

**DOI:** 10.1038/s41598-025-87289-w

**Published:** 2025-03-06

**Authors:** Enzo E. Seculi Pereyra, Juan Vrdoljak, Martín D. Ezcurra, Javier González-Dionis, Carolina Paschetta, Ariel H. Méndez

**Affiliations:** 1 CONICET. Instituto Patagónico de Geología y Paleontología (CCT CONICET CENPAT), Bv. Brown 2915, Puerto Madryn, Chubut 9120 Argentina; 2 CONICET. Instituto Patagónico para el Estudio de los Ecosistemas Continentales, (CCT CONICET CENPAT), Bv. Brown 2915, Puerto Madryn, Chubut 9120 Argentina; 3 CONICET. Sección Paleontología de Vertebrados, (CONICET–Museo Argentino de Ciencias Naturales “Bernardino Rivadavia”), Av. Ángel Gallardo 470, C1405DJR Ciudad Autónoma de Buenos Aires, Buenos Aires, Argentina; 4 School of Geography, Earth and Environmental Sciences, University of Birmingham, Edgbaston, Birmingham, B15 2TT UK; 5 CONICET. Instituto Patagónico de Ciencias Sociales y Humanas “Dra. María Florencia del Castillo Bernal” (CCT CONICET CENPAT), Bv. Brown 2915, Puerto Madryn, Chubut 9120 Argentina; 6 Programa de Referencia y Biobanco Genómico de la Población Argentina, Secretaría de Planeamiento y Políticas en Ciencia, Tecnología e Investigación, Ciudad Autónoma de Buenos Aires, Argentina

**Keywords:** Evolution, Palaeontology

## Abstract

Abelisauridae is a clade of theropods distinguished by short, ornamented skulls and strongly reduced forelimbs. They represented the most abundant predatory dinosaurs in Gondwana during the Cretaceous. Bolstered by biomechanical studies, the morphology of the skull and vertebral column of abelisaurids, have led researchers to hypothesize that Late Cretaceous forms were “specialized hunters.” Here, we use the morphology of the abelisaurid maxilla to test the inclusion of the Lower Cretaceous *Spectrovenator* within the specialized hunter category. Additionally, we analyze the diversity and disparity of the abelisaurid maxilla in a macroevolutionary context. We quantified the maxillary shape in 17 taxa using 2D geometric morphometrics and analyzed different evolutionary scenarios and trends with phylogenetic comparative methods. The results of all the analyses (phylogenetic ordination methods, Z, and R^2^ comparison in phylogenetic generalized least squares, model selection, and estimated taxa-removal analysis) suggest that the hunter specialization appeared during the Early Cretaceous, revealing that Cretaceous abelisaurids can be considered specialist hunters. High levels of morphological disparity in the maxilla occurred shortly after the Cenomanian-Turonian faunistic turnover, which involved drastic changes in the South American terrestrial faunal assemblages. Moreover, the high evolutionary rates of the maxillary shape change in Abelisauridae support a shift in ecological pressures or socio-sexual mechanisms, which were the main drivers of the evolution of the clade rostrum. Our study invites to analyze more osteological elements of the abelisaurid skull under a quantitative macroevolutionary framework to test our results more comprehensively.

**Supplementary Information:**

The online version contains supplementary material available at 10.1038/s41598-025-87289-w.

## Introduction

Non-avian theropod dinosaurs are characterized by a highly diverse morphology^1,2^ and ecology^3–6^. In particular, their skulls possessed an important morphological disparity^2^, which evolved under different evolutionary rates and patterns of phylogenetic modularity and integration^7,8^; although the rostral elements have shown a certain degree of phylogenetic correlation in Archosauria^7^. Revealing these different aspects of the skull evolution enhances our understanding of the macroevolutionary dynamics of extinct taxa.

Abelisauridae is the most abundant and best-known clade of theropod dinosaurs from Gondwana during the Early Jurassic to the end of the Cretaceous^9–11^. Since their recognition almost 40 years ago, members of this group have been identified in various regions of the globe, including India, Madagascar, Africa, southern France, Brazil, and primarily Argentina^4,11^. Many distinctive cranial and postcranial traits characterize abelisaurids and set them apart from other theropod groups^12^. Their skull is typically tall, short, and ornamented, with a highly kinetic intramandibular articulation^3,13,14^. Some anatomical and biomechanical studies of the skull and vertebral column, especially in the cervical area, suggest that Late Cretaceous abelisaurids had a specialized predatory strategy^3,13,14^. This specialized feeding strategy involved primarily the use of the head for hunting, with a short distance sprint, and holding the prey during the kill^3,15^. This strategy has been attributed to taxa with the following features: (1) high bit force, (2) short, broad, and tall skull, (3) short hindlimbs, (4) tendency towards hypermineralization and fusion of skull elements, (5) expanded occiput and neck musculature, and 6) skull resistance to torsional bending^13,14^. On the other hand, a generalist feeding strategy involves strike and tear bites to generate fatal bounds, gradually wearing down the prey to death^3,15^. As a result, taxa with generalized feeding strategies tend to possess well-developed forelimbs^3,15^ and weaker torsional bending resistance during biting^16,17^, as was inferred for the tetanuran *Allosaurus*^18^. However, this hypothesis of predation strategy in Late Cretaceous abelisaurids remains to be tested in a quantitative macroevolutionary framework.

A quantitative macroevolutionary framework allows to understand the phenotypic evolution over time. In this sense, Geometric Morphometrics (GM) quantifies phenotypes in a powerful multivariate way while preserving the geometric structure of the shape^19^ and Phylogenetic Comparative Methods (PCM) offer analytical tools to explore evolutionary modes and tempos quantitatively and in an explicit phylogenetic context in both extant and extinct taxa^20^. GM and PCM can be combined to elucidate how multivariate phenotypes evolved over time^21^. For instance, the combination of these tools has shown complex macroevolutionary dynamics in the cranium of crocodyliforms^22^ and different evolutionary regimes in the evolutionary radiation of hominids^23^. Thus, GM and PCM are important tools for understanding the complex macroevolutionary dynamics of multivariate phenotypes in the Tree of Life.

Abelisaurids represent an excellent opportunity to study the morphological evolution of the skull and individual cranial bones using GM and PCM. The maxilla is well-represented in the abelisaurid fossil record, and because it was involved in the capture and prey processing, it is directly linked to the hypothesized specialized predation strategy. Moreover, morphological changes in maxillary shape (depth and length) correlate with the short and tall skull that characterizes abelisaurids (i.e. abbreviated skull)^3^. This makes the maxilla an excellent osteological element to understand the evolution of the abelisaurid rostrum and skull and test different strategies in this group. Our objective is to characterize the evolution of the maxilla, as a proxy of skull shape, in Abelisauridae and test the correlation of its shape with predation strategies. Additionally, since the description of the Early Cretaceous taxon *Spectrovenator*^4^, the specialized predation strategy hypothesized in Late Cretaceous abelisaurids has gained more support. Thus, we tested the inclusion and similarities of *Spectrovenator* with specialized hunter taxa (= Late Cretaceous abelisaurids). This objective is achieved by quantifying the maxillary shape of abelisaurids and closely related taxa using 2D GM and employing PCM to elucidate: (1) the phylogenetic trends of ecological groups and the position of *Spectrovenator* in the phylogenetics morphospace, (2) whether the shape data supports alternative hypotheses (Posterior hypothesis) that include *Spectrovenator* within the group of specialist hunters, and (3) fitting different evolutionary models to test the Prior (hypothesis that excludes Jurassic and Early Cretaceous taxa from the specialized hunter strategy) and the Posterior hypotheses (determined based on the information provided by the maxillary shape that includes *Spectrovenator* among the specialized hunters). Thus, we aim to explore and determine which model and hypothesis best explains the evolution of the maxilla in Abelisauridae. A brief description of some terms, datasets, and analyses used in this study is provided in Table 1 for a better understanding of the Results and Methods section.

## Results

### Phylogenetic variation and morphological disparity among specialist and generalist taxa

**Fig. 1:**
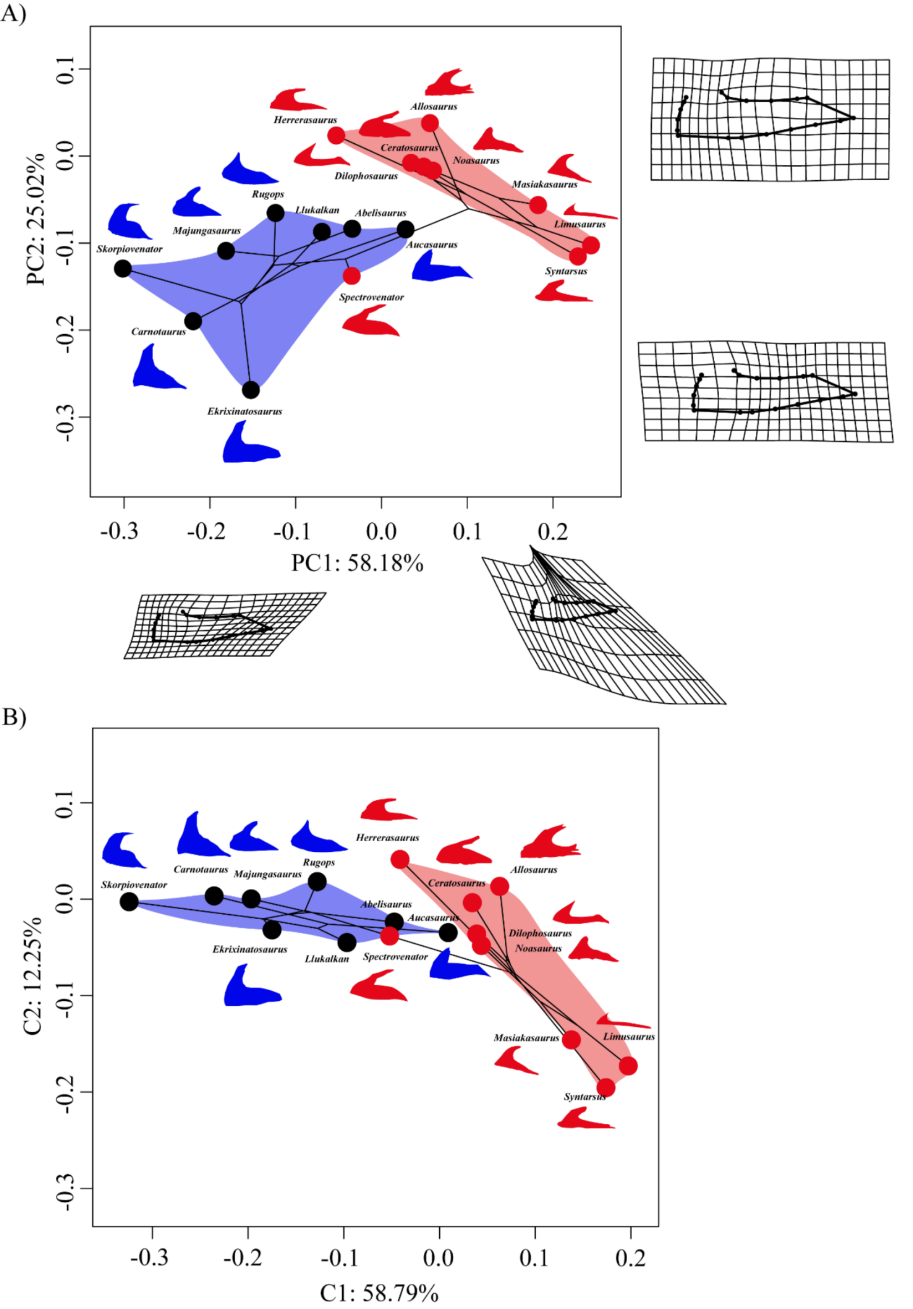
**A** Phylogenetic PCA showing the first two Principal Components (PC) and deformation grids representing the shape of each extreme of the axis. The ratios represent the variance explained by each PC. **B** Phylogenetic aligned component analysis (PACA) showing the first two components (C1 vs. C2). The numbers on the axes represent the percentage of tip dispersion. Black and red points represent specialist and generalist hunters, respectively, according to the Prior hypothesis. The blue areas delimit the morphospace occupied by Abelisauridae and the red areas delimit the morphospace occupied by the outgroups.

The maxillary shape differences were explored in a phylogenetic framework using two phylogenetic ordination methods, Phylogenetic PCA (Phylo-PCA) and Phylogenetic aligned component analysis (PACA). The first two components of the Phylo-PCA explained 83.2% of the total variance of the data (Fig. 1A). The first Phylo-PC was related to the profile of the maxillary body and the elongation of the jugal ramus. This component distinguished Noasauridae and “*Syntarsus*” from Abelisauridae and the rest of the outgroups in the presence of a more curved maxillary body and elongated jugal ramus (Fig. 1A). The phylo-PC2 distinguished the maxillary shape of Abelisauridae from that of *Ceratosaurus*,* Dilophosaurus*,* Herrerasaurus*, and *Allosaurus* in the presence of a broader ascending ramus at the level of the maxillary fenestra in the former taxa. *Carnotaurus*, *Ekrixinatosaurus*, and *Skorpiovenator* showed the most extreme maxillary shapes among Abelisauridae, being particularly short and having a straight maxillary body and a broad maxillary ramus.

The first two principal components of the Phylo-PCA and the PACA did not show a separation between generalist and specialist hunters, although there was a separation at a systematic level (Abelisauridae and outgroups, Fig. 1A and B). The maxillary shape of *Spectrovenator* was recovered within the morphospace occupied by Late Cretaceous abelisaurids. *Aucasaurus*, *Llukalkan*, and *Abelisaurus* were recovered close to the morphospace occupied by the outgroups (= generalist hunters*)*.

**Fig. 2:**
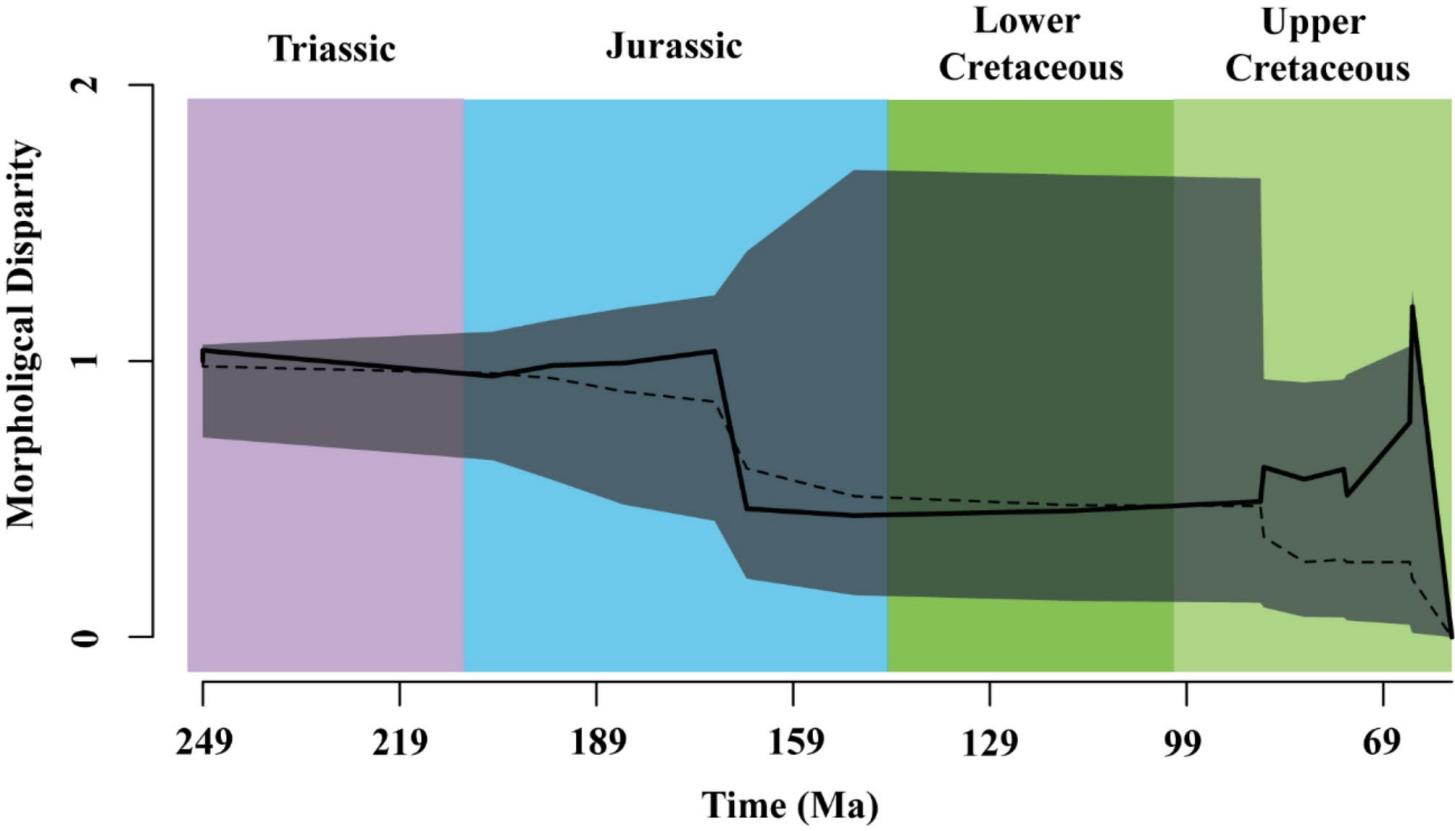
Disparity through time estimated from 90% of the variance of the Phylo-PCs. The solid line represents the observed data, the dashed line represents the mean of simulations under the BM model, and the gray area is the 95% confidence interval of the simulated data set.

The disparity through time plot (dtt), using the 90% of the variances of the Phylo-PCs, was performed to explore the phenotypic variation among abelisaurids. The dtt analysis (Fig. 2) showed a low morphological disparity between ~ 170 Ma and ~ 90 Ma (late Early Jurassic–early Late Cretaceous). Although the dtt analysis suggests a Brownian motion model for the evolution of the maxilla, an increase in disparity was observed in the abelisaurid maxilla between ~ 90 Ma and 66 Ma (late Upper Cretaceous).

**Fig. 3:**
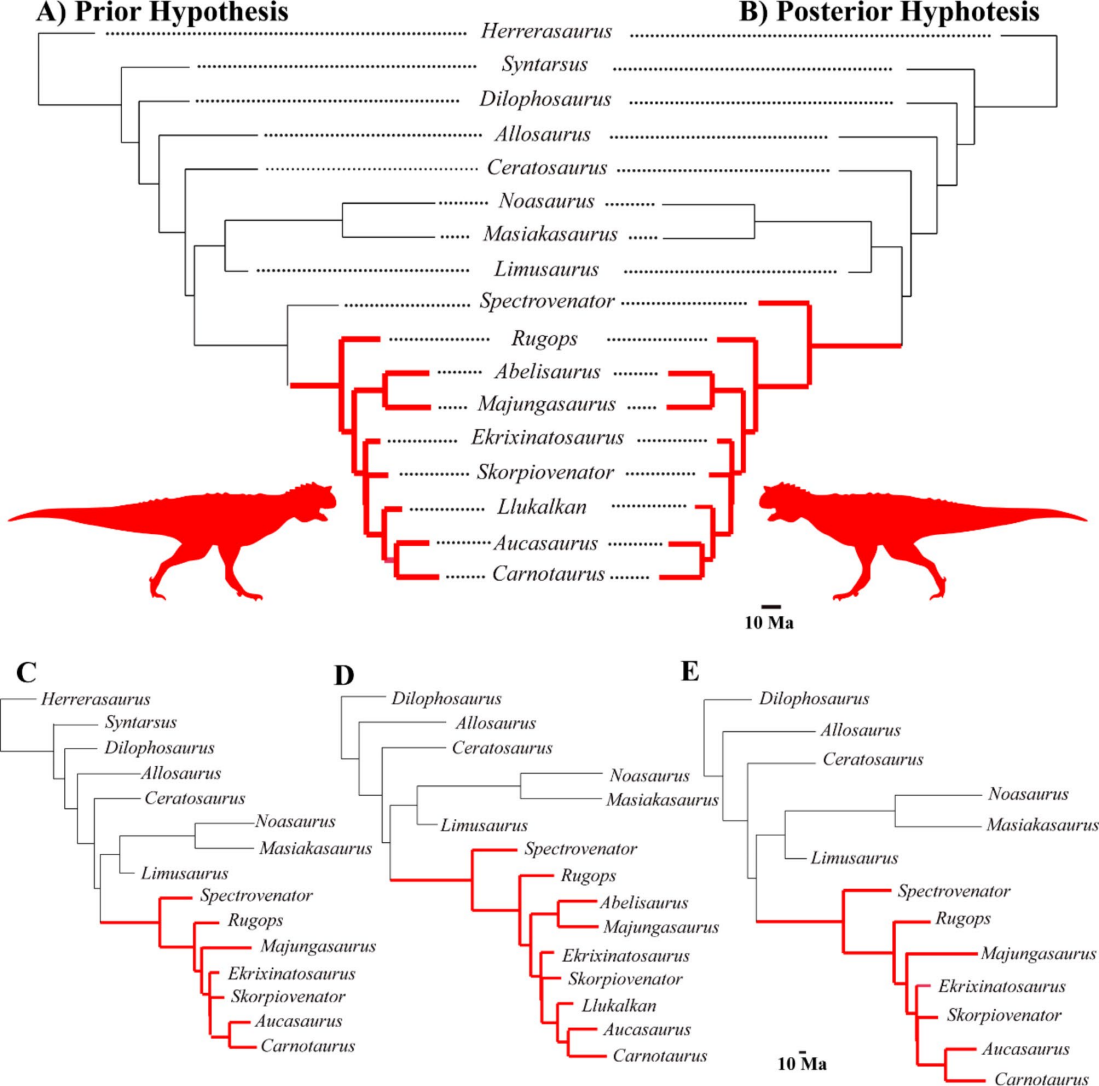
Hypothesis of type of predation strategy evolution (black “generalist hunters” and red “specialist hunters”). **A** Prior Hypothesis; **B** Posterior Hypothesis obtained in the SURFACE analysis with “all taxa clade”; **C** Posterior Hypothesis obtained in the SURFACE estimated taxa-removal analysis with “all taxa clade”, **D** Posterior Hypothesis obtained in the SURFACE analysis with the “Dilophosaurus clade”, and **E** Posterior Hypothesis obtained in the SURFACE estimated taxa-removal analysis with the “Dilophosaurus clade”. *Carnotaurus* silhouettes from PhyloPic (http://phylopic.org).

### Evolutionary pattern without prior hypothesis

We analyzed whether the shape data supports alternative hypotheses to that of the Prior hypothesis (Fig. 3A) for the evolution of predation strategy in Abelisauridae. All the SURFACE analyses detected the same optima in the Abelisauridae phylogeny (Fig. 3B–E), indicating that this clade represents a phenotypic optimum regardless of the outgroup sampling (see Methods, evolutionary models and estimated taxa-removal analysis). We called this new hypothesis, where *Spectrovenator* is considered a specialist hunter, as the Posterior hypothesis.

### Phylogenetic signal and phylogenetic generalized least square (PGLS) regressions

A phylogenetic signal analysis to assess the evolutionary structure of the maxillary shape was performed using Procrustes Coordinates (PC), Phylo-PCA, and PACA. The *Kmult* value of each dataset showed no evidence of a significant phylogenetic signal for the maxillary shape (Table 2).

**Fig. 4:**
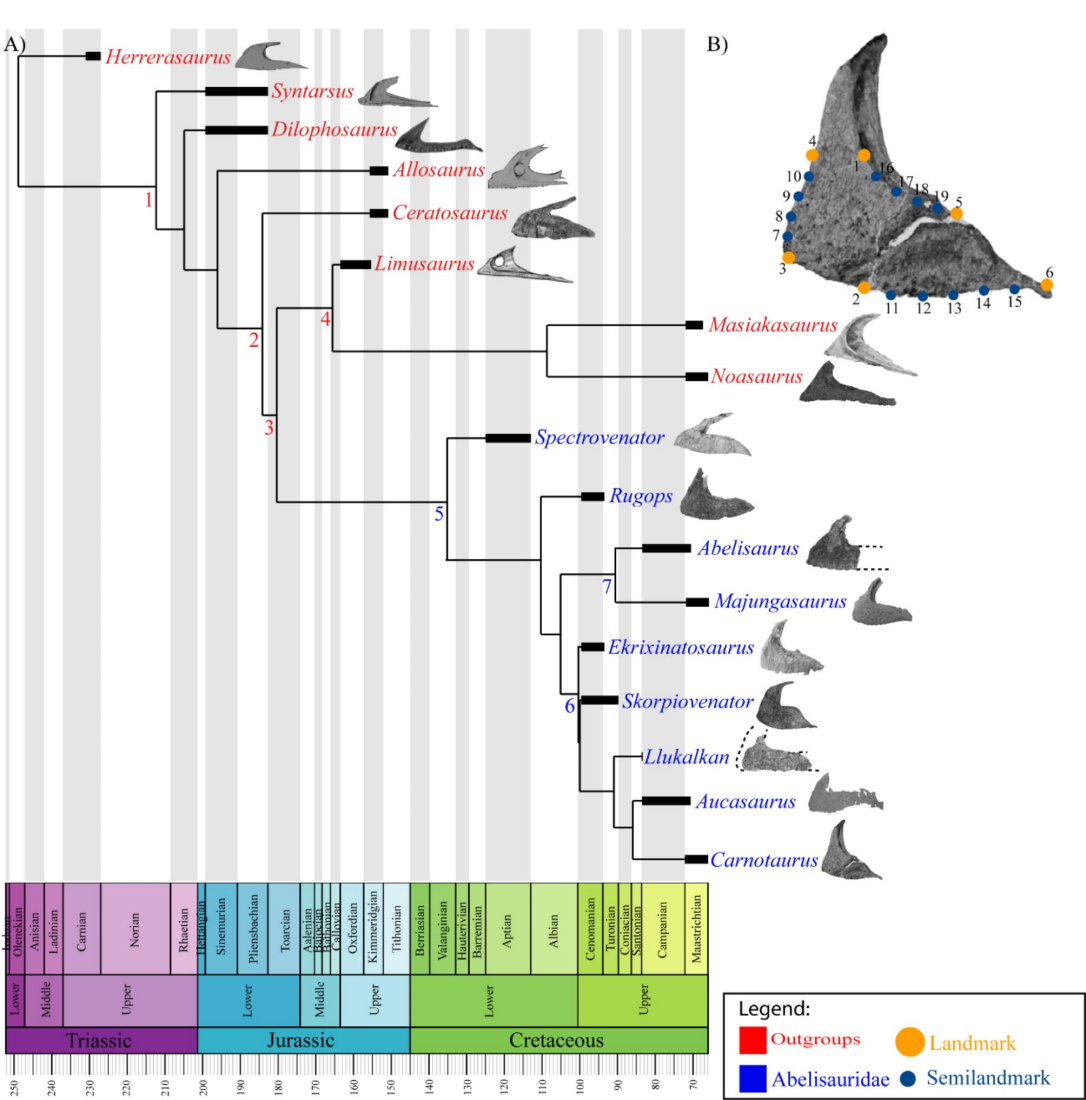
**A** Time-calibrated global reduced strict consensus tree used in our analyses showing the abelisaurids (blue) and outgroups (red) that preserve maxilla. The dashed line in some maxillae denotes an incomplete element. Nodes: (1) Neotheropoda, (2) Ceratosauria, (3) Abelisauroidea, (4) Noasauridae, (5) Abelisauridae, (6) Brachyrostra and (7) Majungasaurinae. **B** Landmark configuration over *Carnotaurus. * (modified from Cerroni et al., 2021^51^) used in the analyses, in which landmarks are in yellow and semilandmarks are in blue.

**Table 1:** Brief descriptions of the datasets, terms, and analyses used in this study.

Dataset/term/ analysis	Brief Description
Specialist Hunters	Taxa with a specialist predation strategy
Generalist Hunters	Taxa with a generalist predation strategy, which include the phylogenetic outgroups of the Abelisauridae tree and *Spectrovenator* in the Prior hypothesis
Prior Hypothesis	Hypothesis proposed in previous studies, stating that Late Cretaceous abelisaurids had a specialist predation strategy
Posterior Hypothesis	Hypothesis obtained in the SURFACE analysis, in which, without a Prior hypothesis, one or more shifts might be obtained in the abelisaurid phylogeny using the Phylo-PCA data. This hypothesis includes the Early Cretaceous *Spectrovenator* inside of the specialist hunters group
All taxa clade	Abelisauridae phylogeny in which all taxa were considered for the evolutionary model analyses and the evolutionary hypothesis without a Prior hypothesis
Dilophosaurus clade	Abelisauridae phylogeny in which the earliest-diverging taxa (*Herrerasaurus* and *Syntarsus*) were removed before the analysis
Estimated taxa	Taxa with estimated landmark coordinates (*Lukalkan* and *Abelisaurus*)
Estimated taxa-removal analysis	Analysis performed with the aim to evaluate how the results vary when estimated taxa are removed from the evolutionary model analyses and the evolutionary hypothesis without a Prior hypothesis analysis

**Table 2:** Phylogenetic signal analysis using Procrustes coordinates (PC), 90% of tip dispersion in phylogenetic PCA (Phylo-PCA), and 90% of tip dispersion in phylogenetic aligned components analysis (PACA).

Data	Scaling Lambda	Log-likelihood	Z	Kmult	*p*-value
PC	0	-	-	-	-
90% PhyloPCA (1–4)	1	52.40	0.02	0.31	0.49
90% PACA (1–5)	0.1	138.10	0.97	0.32	0.16

Lambda for tree scaling (scaling lambda), effect size (Z), multivariate K static (Kmult), and p-values calculated under permutation procedure. When Lambda scaling is 0 there is no phylogenetic signal in the data and values of Log-Likelihood, Z, *Kmult*, and p-value are not returned in the analyses.

The presence of allometry in the shape of the maxilla and the correlation with the type of predation strategy (TPS) were tested in a quantitative evolutionary framework using PGLS regressions in the Prior and Posterior hypothesis and then effect size (Z) and the variances explained for the factor (R^2^) were compared between hypotheses. We found that the maxillary size (centroid size) did not have an evolutionary significant effect on the shape of the bone in all datasets: PC, Phylo-PCA, and PACA (see Supplementary Data 1: Table 3). Moreover, the interaction between factors (TPS and centroid size) in all datasets explained around 10% of the total variation (R^2^ = ~ 0.1; see Supplementary Data 1: Table 3). However, the TPS explained more than 40% of the total variation of the maxillary shape in Phylo-PCA, and PACA (R^2^ = ~ 0.44; see Supplementary Data 1: Tables 4, 5, 6 and 8–9). Regarding the Z and R^2^ comparison between hypotheses, except for the PC analysis, the Z and R^2^ values for the TPS were higher in the Posterior than the Prior hypothesis (see Supplementary Data 1: Table 3).

### Evolutionary models (prior vs. posterior hypothesis), estimated taxa-removal analysis, and model adequacy

Different evolutionary models were fitted in the Prior and Posterior hypotheses with the aim to explore and determine which model and hypothesis best explains the evolution of the maxilla of Late Cretaceous abelisaurids as “specialist hunters”. The model comparisons showed that a two-rate Brownian motion model (BMM) under the Prior hypothesis was the model with the best fit (Table 3). Regarding the estimated evolutionary rates of this model, all the parameters were almost similar between specialist and generalist hunters (see Supplementary Data 1: Table 4).

In the “Dilophosaurus clade” (see Table 1 for a definition of this group) the BMM for the Posterior hypothesis was the best-supported model (Table 3). Regarding the evolutionary rate matrix estimated by BMM, the evolutionary rates were higher in specialist than generalist hunters, especially in Phylo PC1 and Phylo PC2 (see Supplementary Data 1: Table 5).

**Table 3:** Evolutionary models fitted in “All taxa clade” and “Dilophosaurus Clade” using the 90% of Phylo-PCs with log-likelihood (L), Akaike Information Criterion (AIC), and AIC weight values (AICw).

	Model	L	AIC	AICw		L	AIC	AICw
All taxa clade	OU1	102.59	-200.55	0.00	Dilophosaurus clade	97.91	-139.83	0.02
	OUM	105.57	-196.18	0.00		98.93	-133.86	0.00
	BM	86.29	-202.83	0.01		78.66	-129.32	0.00
	**BMM**	**96.44**	**-211.11**	**0.84**		95.21	-142.41	0.07
	EB	87.29	-186.08	0.00		78.68	-127.36	0.00
	BMMS	96.36	-207.55	0.14		**97.73**	**-147.47**	**0.90**
	OUMS	103.21	-194.74	0.00		101.29	-138.57	0.01

OU1: single phenotypic optimum, OUM: two phenotypic optima fitted in the prior hypothesis, BM: single rate Brownian motion, BMM: two-rates Brownian motion fitted in the prior hypothesis, EB: early burst, BMMS: two rates brownian motion fitted to the posterior hypothesis, and OUMS: two phenotypic optima fitted to the posterior hypothesis. Bold numbers represent the best model/s.

**Table 4:** Evolutionary models fitted in estimated taxa-removal analysis in “All taxa clade” and “Dilophosaurus Clade” using the 90% of Phylo-PCs with log-likelihood (L), Akaike Information Criterion (AIC) and AIC weight values (AICw).

	Model	L	AIC	AICw		L	AIC	AICw
All taxa clade	OU1	102.48	-148.96	0.00	Dilophosaurus clade	54.66	-73.33	0.00
	OUM	106.12	-148.23	0.00		58.51	-75.01	0.00
	BM	73.59	-119.18	0.00		39.66	-61.32	0.00
	BMM	105.77	-163.53	0.26		63.66	-97.32	0.08
	EB	80.86	-131.73	0.00		45.26	-70.51	0.00
	**BMMS**	**106.83**	**-165.67**	**0.74**		**66.13**	**-102.25**	**0.92**
	OUMS	107.92	-151.83	0.00		59.67	-77.34	0.00

OU1: single phenotypic optimum, OUM: two phenotypic optima fitted in the prior hypothesis, BM: single rate Brownian motion, BMM: two-rates Brownian motion fitted in the prior hypothesis, EB: early burst, BMMS: two rates brownian motion fitted to the posterior hypothesis, and OUMS: two phenotypic optima fitted to the posterior hypothesis. Bold numbers represent the best model/s.

Given that estimated landmark coordinates might bias the results, we removed taxa with estimated missing landmarks and re-evaluated the evolutionary models in both Prior and Posterior hypotheses (“estimated taxa-removal”). The BMM was the best-supported model under the Posterior hypothesis (Table 4) in “All taxa clade” and “Dilophosaurus clade”. The evolutionary rate matrix estimated by the BMM models in “All taxa clade” and “Dilophosaurus clade” showed that the evolutionary rates were higher in specialist hunters than generalist hunters (see Supplementary Data 1: Table 6, and 7).

The diagonal parameter matrix values of the best models in each data set (“All taxa clade”, “Dilophosaurus clade” and “estimated taxa-removal”) were within the 95% confidence interval of each parameter (see Supplementary Data 1: Table 8, and 9), suggesting the adequacy of the models.

## Discussion

The Prior hypothesis proposes that Late Cretaceous abelisaurids had a specialist feeding strategy^3,4,13,14,24,12,19^. Because the maxilla is correlated with skull features associated with the predation strategies hypothesized for Late Cretaceous abelsiaurids^3^ and is directly involved in the capture and prey processing, this bone is expected to have been strongly influenced by ecological pressures in Late Cretaceous abelisaurids. The phylogenetic ordination methods revealed that the maxilla of the Early Cretaceous abelisaurid *Spectrovenator* is morphologically closer to that of more deeply nested abelisaurids than the outgroups. Z scores and R^2^ values were the highest in the Posterior hypothesis in PGLS analysis. Additionally, when abelisaurids were investigated deeper in the phylogeny, some outgroups were excluded from the analysis, the best model supported by data was the multi-rate Brownian motion under the Posterior Hypothesis. Furthermore, Early Cretaceous abelisaurids likely had the specialized predations strategy hypothesized for Late Cretaceous abelisaurids. These results stand in the estimated taxa-removal analyses. Indeed, five arguments derived from our results support the fact that the shape of the maxilla in Cretaceous abelisaurids is unique among our sample and evolved under different evolutionary rates: (1) the maxilla of the Early Cretaceous abelisaurid *Spectrovenator* was more similar to that of Late Cretaceous abelisaurids than to the outgroup taxa in the phylogenetic ordination methods; (2) Z and R^2^ values were higher in the PGLS analysis under the Posterior Hypothesis; (3) in the model selection analysis, when outgroups were pruned (see Methods), the multi-rate Brownian motion under the Posterior hypothesis best fitted our data; (4) all of the estimated taxa-removal analyses indicated two evolutionary rates Brownian motion models under the Posterior hypothesis; and 5) the confidence intervals showed adequacy of the best models to the Posterior hypothesis. This scenario is congruent with the Posterior hypothesis.

Regarding the evolution of the maxilla, a multi-rate Brownian motion was the best-supported evolutionary model in the Posterior hypothesis. This multi-rate Brownian motion model can be interpreted as a first approximation with the currently available data because our sample lacks abelisaurid species without a preserved maxilla. Evolutionary rates were higher among “specialist hunters” (= Abelisauridae) than in “generalist hunters” (outgroup taxa), suggesting that the abelisaurid maxilla evolved comparatively faster. Our results agree with those recently obtained by Pol et al. (2024)^25^, in which high cranial evolutionary rates were calculated at or around the base of the clade that includes Cretaceous abelisaurids based on the analysis of a matrix composed of discrete characters. Felice et al. (2020)^7^ proposed that the cranial vault and the relative position of the jaw joint experienced a rapid evolution in non-avian dinosaurs. They linked this difference in rates to bony ornamental structures, food acquisition strategies, and diets. Ornamental structures might evolve through species recognition or sexual selection, and it is expected to stay under selection and show high rates of evolution^7,26^. In this sense, the PGLS and evolutionary models showed that the shape of the abelisaurid maxilla was linked to the type of predation strategy, which evolved under high evolutionary rates. Additionally, the abelisaurid maxilla has different ornaments, with rugosities being a distinctive feature differentiating members of this clade from non-abelisaurid theropods. In this sense, the shape of the abelisaurid maxilla bolstered a shift in ecological pressure or socio-sexual mechanism in non-avian theropods, which agrees with that suggested by previous studies on skull morphology^7^ and body size^27^. However, the evolution of the ornamentation in Abelisauridae has to be studied using a broader sample of skeletal elements (e.g. skull roof bones) in an explicit, quantitative macroevolutionary context. Moreover, the rostral elements of non-avian dinosaurs are evolutionary correlated^8^ and, therefore, the abelisaurid premaxilla and nasal could exhibit the same evolutionary signal and have evolved under similar evolutionary pressures.

The dtt showed low levels of disparity in Abelisauridae between the Middle Jurassic and the early Late Cretaceous. This result is likely related to the scarce record of abelisaurids in this time range (*Spectrovenator* is the only sampled abelisaurid) and an improved sampling is necessary to test if it is a bias or an actual biological pattern. Abelisaurids reached a peak of morphological disparity at the end of the Cretaceous before the mass extinction. It is interesting to note that the increase in disparity in Abelisauridae started slightly after the proposed Cenomanian-Turonian faunistic turnover, which is characterized by the disappearance of carcharodontosaurid theropods and rebbachisaurid sauropods^11^. Some researchers have claimed that Abelisauridae diversified and occupied ecological niches left vacant by carcharodontosaurids^11,28–30^, but this hypothesis remains to be tested quantitatively. The dtt analysis of the maxillary shape indicates that Abelisauridae, along with megaraptorids, diversified and probably occupied ecological niches left vacant by the carcharodontosaurids following their extinction in the Turonian. A possible explanation for these high levels of disparity is that abelisaurids had a greater ability to adapt to changing environments than other medium and large-sized theropods, which prompted their diversification during the Late Cretaceous in Gondwana.

Brusatte et al. (2012)^2^ hypothesized that in deeper lineages of non-avian theropods the phylogenetic signal could disappear because sexual selection and ecological pressures on clades might have had more influence than the phylogenetic relationships between species. Tse et al. (2024)^31^ detected a weak phylogenetic signal in the dromaeosaurid skull, matching the claim that the phylogenetic signal disappears in deeper linages of non-avian theropods. The results of maxillary shape were consistent with these observations as we did not find any evidence of a significant phylogenetic signal in the abelisaurid maxilla. Also, the PGLS analyses showed that the type of predation strategy influenced more the evolution of the maxilla than size. Regarding this, Zaher et al. (2020)^4^ suggested that body size influenced the highly modified feeding strategy hypothesized for Late Cretaceous abelisaurids. However, our results indicate that the type of predation strategy better explains the maxillary shape than the size when *Spectrovenator* is also considered a specialist hunter (Posterior Hypothesis).

In addition, since the studies of Mazzeta et al. (1998)^13^ and Therrien et al. (2005)^14^, no new biomechanical studies were conducted on the abelisaurid jaw. Biomechanical data can be combined with PCM to elucidate the palaeoecology and macroevolution of Abelisauridae. Encompassing how different osteological elements evolved among abelisaurids in macroevolutionary frameworks would help to understand how this successful group flourished during the Cretaceous in the Gondwanan landmasses.

In conclusion, this study, which evaluated the evolution of the maxillary shape and tested hypotheses of hunting strategy in abelisaurids, suggests that Cretaceous species were specialized predators. We additionally showed that the diversity and disparity of the abelisaurid maxilla exhibit signals of ecological pressures, while size did not significantly influence the evolution of the specialized predation strategy in Abelisauridae. Our study invites to analyze more osteological elements of the abelisaurid skull and their post-cranial skeleton under a quantitative macroevolutionary framework to test our results more comprehensively.

## Methods

### Phylogenetic hypothesis

We used the phylogenetic data matrix of Pol et al. (2024)^25^, without modifications but with a different tree search protocol, to generate an updated hypothesis of the relationships among abelisaurid theropods. The dataset was analyzed under maximum parsimony with implied weighting. The decision of weighting against homoplasy relies on the results and recommendations of Goloboff et al. (2008, 2018)^32,33^, based on simulation-based analyses, and Ezcurra (2024)^34^, based on analyses on empirical data, who recovered that implied weighting outperforms equal weighting in both accuracy and precision. We used concavity constants (k) between 3 and 8 (i.e., a total of six analyses) because this range was shown to provide more stable results in analyses of empirical phylogenetic genealogies with a number of terminals similar to that of our dataset^34^. The search for the most parsimonious trees (MPTs) followed a “traditional search” method, using 1,000 replications of Wagner trees (with random addition sequence) followed by TBR branch swapping (holding 10 trees per replicate). A second round of TBR branch swapping was conducted on the trees retained in memory. Consistency (CI) and retention (RI) indices were calculated using the script (STATSb.run) published by Spiekman et al. (2021)^35^, which does not include inactivated terminals in the calculations. Topologically unstable terminals among the MPTs of each analysis were detected with the iterPCR protocol^36^ and removed a posteriori (Supplementary Data 5) to generate a global reduced strict consensus tree (GRSCT) from all the MPTs found in the six analyses with different k values. This GRSCT was used for all subsequent analyses. The phylogenetic analyses were conducted in TNT v. 1.6^37^ using a custom script written for this software (Supplementary Data 10).

### Tree temporal calibration

The comparative phylogenetic methods used here require a time-calibrated phylogenetic tree. As a result, the GRSCT was time-calibrated with the stochastic cal3 method implemented in the R package paleotree^38^. The range of chronostratigraphic uncertainty of each species included in the tree was obtained from the Paleobiology Database (https://paleobiodb.org; accessed June 2024) (Supplementary Data 2–3). Each calculation based on a different set of branch, extinction, and sampling rates retained 10 trees, which resulted in a total of 780 time-calibrated trees. We generated a final tree with mean branch lengths using the function *consensus.edges* of the phytools package^39^, and all zero-length branches were replaced with 0.1 million years (Fig. 4A) (Suplementary Data 6 & 8).

### Sampled data, geometric morphometrics, and predation strategy coding

We used 2D published maxilla images of abelisaurids and closely related taxa that were sampled in the phylogenetic matrix of Pol et al. (2024)^25^ (*Koleken incalayaly* was not used in the analysis because it was published after we conducted our analyses). The maxilla of a total of nine abelisaurid and eight closely related outgroup species were used in the analyses (see Supplementary Data, Table 1). There are more abelisaurid maxillae in the fossil record (e.g. *Tralkasaurus*, *Kryptops*, and *Rajasaurus*), but they were not considered here because of their extremely fragmentary nature, unstable phylogenetic position, or their absence in quantitative phylogenies. We used the left maxilla in our analyses, but when only the right element was available, we mirrored the image to allow landmarking.

We designed a 2D configuration with 6 landmarks and 13 semilandmarks, digitized in tpsDig (2.6.4)^28^ (Fig. 4B and Supplementary Data 1: Table 2; Landmark Data, Supplementary Data 2), considering that 1) the number of landmarks are constrained by the number of species (it is usually recommended the presence of more species than landmarks), which affects downstream analyses^21^, and 2) that the landmark configuration correctly samples shape changes related to the observed morphological features in the abelisaurid skull (mainly related to length and height). Since the complete maxillae of *Llukalkan* and *Abelisaurus* were unavailable, we estimated the missing landmarks using the thin plate spline method^40^ (*estimate.missing*, geomorph package v.4.0.7^41^). Finally, a Generalized Procrustes Analysis was performed to eliminate the effects of rotation, translocation, and scale, obtaining the shape and size variables that were used for downstream analyses (*gpagen*, geomorph package^41^).

Different studies have claimed that Late Cretaceous abelisaurids had a specialized feeding strategy. Thus, non-Late Cretaceous abelisaurids (i.e. *Spectroveator* and phylogenetic outgroups) were classified as generalists for hypothesis testing. This taxa classification could change based on the results of our analyses, i.e. Posterior hypothesis (see below ‘Evolutionary hypothesis without a Prior Hypothesis’ section).

### Phylogenetic comparative methods

#### Phylogenetic ordination methods and disparity through time

Phylogenetic ordination methods allow the detection of different trends in phenotypic evolution^21^. Trends in the evolution of the abelisaurid maxilla were visualized using the first two components of a Phylogenetic Principal Components Analysis (Phylo-PCA) and Phylogenetic Aligned Components Analysis (PACA). The Phylo-PCA minimizes the phylogenetic variation along the first Principal Components^21^, while the PACA fits most of the phylogenetic variation in the first principal components^42^ (*gm.prcomp*, geomorph package^41^). We follow the maxillary anatomical terminology proposed by Hendrickx and Mateus (2014)^43^.The 90% tip dispersion of variance explained by the phylogenetic ordination methods were used in the subsequent phylogenetic comparative analyses. Thus, when a reference is made to the Phylo-PCA and PACA will be corresponding to 90% of the total variation (Phylo PCs 1–4) or the variation of the tips (PACAs 1–4).

A disparity-through-time plot (dtt) was performed to visualize patterns of shape changes within and among clades^44^ in the abelisaurid phylogeny (*dtt* function, geiger package^45^, v2.0.11). In this method, the disparity is estimated for all taxa of the phylogeny and subsequently for each subclade. Relative disparity is obtained by dividing each subclade disparity value by the total disparity of the clade. Finally, the average relative disparity is estimated for each subclade present at the time of each divergence time^44^.

### Evolutionary hypothesis without a prior hypothesis

We explored the number of evolutionary shifts in Abelisauridae using the 90% of Phylo PCA and the stepwise Akaike Information Criterion calculated by the *surfaceforwad* function of the SURFACE package^46^ (v 0.5). Our goal was to reduce the dimensionality in landmark data with Phylo PCA components and determine if an alternative hypothesis (Posterior hypothesis) could better explain the hunter specialization of the Abelisauridae. This method allows us to estimate an evolutionary adaptive landscape for our data, conveying selective regimes to the branches of a tree^47^. We repeated the SURFACE analyses after removing the estimated taxa and also for the “Dilophosaurus clade” (see Evolutionary models and estimated taxa-removal analysis below).

### Phylogenetic signal (PS) and Phylogenetic Generalized Least Square (PGLS) regressions

We tested the phylogenetic signal (*physignal.z*, geomorh package) of the Procrustes Coordinates that describe the shape of the maxilla using *Kmult*^21^ in the Procrustes Coordinates (PC), Phylo-PCA, and PACA. The PC represents all the variation in maxilla shape data but in order to avoid more variables (numbers of landmarks) than species in the analysis, we also used the components of Phylo-PCA and PACA. PACA maximizes phylogenetic signal in the first axes and Phylo PCA finds principal components that are more related with other source of trait variation (ecological) than phylogeny^21^. Thus, both phylogenetic ordination data are adequate to test phylogenetic signal and further study the relationship between factors (ecological, behavioral, geographic, etc.) and shape in a phylogenetic context. Phylogenetic signal analysis measures the tendency of related species to be more similar in shape than expected by a Brownian motion evolutionary model^21^. Additionally, we studied the relationship between phylogeny, allometry and type of predation strategy (Specialist versus Generalist hunters). We conducted a PGLS on PC, Phylo-PCA, and PACA using the following two cross-factors: type of predation strategy (TPS) and centroid size (as a proxy for the total size of the structure). In this analysis, PACA was used with the aim to test whether the factors used might explain the differences in shape in a phylogenetic ordination method, which mainly shows changes associated with phylogenetic signal^42^. Therefore, if ecological factors explain the difference in shape in PACA, it means that phylogeny did not have a major influence in phenotypic evolution. Subsequently, we reclassified the TPS following the Posterior hypothesis and repeated the PGLS analyses to compare the relationship between the effect size (Z) and the explained variance (R^2^) of the TPS in the Prior and Posterior hypotheses.

### Evolutionary models (prior hypothesis versus posterior hypothesis)

Macroevolutionary models can be used to describe how different phenotypes evolved through time^47^. To explore and determine which model best explains the evolution of the maxilla in Late Cretaceous abelisaurids under the Prior hypothesis, we fitted five evolutionary models to the Phylo-PCA maxilla data on the Abelisauridae phylogeny (“All taxa clade”) using the mvMORPH^48^ package v1.1.9. Phylo PCA was used instead of PACA because Phylo PCA is more related with the type of predation strategy tested in this work. The fitted models were: (1) single evolutionary rate (single Brownian motion (BM), *mvBM* function); (2) two evolutionary rates (multiple Brownian motion (BMM), *mvBM* function); (3) a single phenotypic optimum (OU1, *mvOU* function); (4) two phenotypic optima (OUM, *mvOU* function); and (5) rapid evolution in the early stage of phylogeny (Early burst (EB), *mvEB* function). The overall shape of the maxilla of *Herrerasaurus* and “*Syntarsus*”, which are the two earliest-diverging taxa of our phylogeny, departs considerably from that of tetanuran and ceratosaurian theropods. Their morphology represents the retention of character states plesiomorphic for Saurischia and Neotheropoda, respectively. These features can be misinterpreted in our analysis as a specialization, although they represent plesiomorphies because of the poor sample of species for this part of the non-averostran dinosaur tree. Thus, in order to avoid this possible bias, we tested the evolutionary models after the exclusion of *Herrerasaurus* and *“Syntarsus”* (this reduced data set is referred here to as the “Dilophosaurus clade”, notice that a new Phylo-PCA was performed). “Generalist” and “Specialist” traits were mapped in the abelisaurid tree using the *make.simmap* function (phytools package^39^, 2.1-2) with equal rates. Finally, we tested the fit of BMM and OUM in the Posterior hypothesis obtained from the SURFACE analyses in the “All taxa clade” and the “Dilophosaurus clade” taxonomic samples (notice that OU, BM and EB models do not calculate parameters of the characters mapped in a phylogeny, so that they represent common models between hypotheses). Subsequently, we compared the fit of each model to the Prior and Posterior Hypotheses using Akaike Criterion Information weights (AICw). The R script of the Macroevolutionary analysis can be found in the Supplementary Data 7.

### Estimated taxa-removal analysis and model adequacy

To test the effect of the estimated landmarks on the fit of the evolutionary models, we removed taxa that lacked a complete landmark configuration and re-analyzed the fit of the evolutionary models to both Prior and Posterior hypotheses (estimated taxa-removal analysis). We evaluated whether the best model changed or remained consistent in these analyses. Additionally, ordination reduction methods might produce biased results in model selection^49,50^. To further avoid model misassignments, we performed a simulation analysis to evaluate the adequacy of the best models in the “All taxa clade” and the “Dilophosaurus clade” datasets. We repeated these tests for the best models found in the estimated taxa-removal analysis. We simulated 500 datasets under the best model and conducted 95% confidence intervals for the diagonal parameter matrix that represent the parameters for each Phylo-PC of each model. A model was considered adequate if each parameter fell within its respective confidence interval. The R script of the simulation analysis can be found in Supplementary Data 9.

## Electronic supplementary material

Below is the link to the electronic supplementary material.

Supplementary Material 1

Supplementary Material 2

Supplementary Material 3

Supplementary Material 4

Supplementary Material 5

Supplementary Material 6

Supplementary Material 7

Supplementary Material 8

Supplementary Material 9

Supplementary Material 10

## Data Availability

All data generated or analysed during this study are included in this published article and its supplementary information files as follows: references of maxillary photographs or schemes used, anatomical landmarks description (SP1), landmark data in .tps format (SP2), time intervals (SP3) and FAD-LAD data (SP4), posteriori taxa pruned (SP5), time-calibrated global reduced strict consensus tree (SP6), R scripts of Macroevolutionary analysis (SP7), tree calibration (SP8) and simulation (SP9) analysis, and TNT script (SP10) are available in the Supplementary data files.
