# Supplementary material for: Morphology of the maxilla informs about the type of predation strategy in the evolution of Abelisauridae (Dinosauria: Theropoda)
Source: Sci Rep. 2025 Mar 6;15:7857. doi: 10.1038/s41598-025-87289-w (PMC11885552; doi:10.1038/s41598-025-87289-w)
Supplement: Supplementary file 1 — Supplementary Material 1 [file 41598_2025_87289_MOESM1_ESM.pdf]

Morphology of the maxilla reveals the type of predation strategy in the evolution of the Abelisauridae (Dinosauria: Theropoda)

Enzo E. Seculi Pereyra[1,*], Juan E. Vrdlojak[2,++], Martin D. Ezcurra[3,++], Javier Gonzales Dionis[1,++], Carolina Paschetta[4,+], and Ariel H. Méndez[1,+]

Supplementary material

Supplementary table 1: Literature used to perform the morphometric analysis on the maxilla.

Species	Reference
Aucasaurus	Matias Baiano thesis 2021[1]
Abelisaurus	Matias Baiano thesis 2021[1]
Allosaurus	Emily J. Rayfield *et al* 2001[2]
Carnotaurus	M.A. Cerroni , J. I. Canale & F. E. Novas 2020[3]
Ceratosaurus	Scott D. Sampson & Lawrence M. Witmer 2007[4]
Dilophosaurus	Adam D. Marsh and Timothy B. Rowe 2020[5]
Herrerasaurus	Paul C. Sereno & Fernando E. Novas [6]
Majungasaurus	Scott D. Sampson & Lawrence M. Witmer 2007[4]
Masiakasaurus	Rafael Delcourt 2018[7]
Rugops	Paul C. Sereno, Jeffrey A. Wilson and Jack L. Conrad 2004[8]
Skorpiovenator	Juan I. Canale, Carlos A. Scanferla, Federico L. Agnolin & Fernando E. Novas 2009[9]
Spectrovenator	Hussam Zaher, Diego Pol, Bruno A. Navarro, Rafael Delcourt and Alberto B. Carvalho 2020[10]
Ekrixinatosaurus	Matias Baiano thesis 2021[1]
Llukalkan	Federico A. Gianechini, Ariel H. Méndez, Leonardo S. Filippi, Ariana Paulina-Carabajal, Rubén D. Juárez-Valieri & Alberto C. Garrido 2021[11]
Syntarsus	Timothy Rowe 1989[12]
Limusaurus	Xing Xu *et al* 2009[13]
Noasaurus	Rafael Delcourt 2018[7]

Supplementary table 2: Anatomical landmarks description of each landmark in Figure 4 B. * Denote semilandmarks

Landmarks	Anatomical description
1	Dorsal margin of the maxillary border at the posterior limit of the external contour of the maxilla
2	Ventral margin of the maxilla directly beneath landmark 1
3	Antero-ventral corner of the maxillary body
4	Anterior maxillary margin in front of point 1
5	Dorsal margin of the external maxillary surface at anterior limit of jugal contact
6	Postero-ventral corner of the maxilla at the jugal contact surface
7-10*	Anterior maxillary border between landmark 3 and 4
11-15*	Ventral maxillary border between landmark 2 and 6
16-19*	Dorsal margin of maxillary border between landmark 1 and 5

Supplementary table 3: Phylogenetic generalized least square in Procrustes coordinates, 90% Phylo PCA and 90% PACA in Prior and Posterior hypothesis. Variables: TPS (Type of predation strategy: Specialist hunters and Generalist hunters), Size of maxilla and the interaction between the TPS and size (TPS*Size). DF: freedom degrees, SS: sum of square. R^2: Variance explained by the factor in data. Z: effect of size. In bold significant effects (p-value <0.05).

Variables	Data	Prior hypothesis				Posterior hypothesis			
		Df	SS	R^2	Z	Df	SS	R^2	Z
Size	PC	1	0.001	0.03	-0.48	1	0.002	0.06	0.39
TPS	PC	1	0.001	0.06	0.15	1	0.001	0.05	0.13
TPS*Size	PC	1	0.004	0.14	1.57	1	0.003	0.14	1.68
Residuals	PC	13	0.020			13	0.019	0.75	
		DF	SS	R^2	Z	Df	SS	R^2	Z
Size	Phylo PCA	1	0.013	0.03	0.11	1	0.013	0.03	0.29
TPS	Phylo PCA	**1**	**0.212**	**0.43**	**3.07**	**1**	**0.234**	**0.47**	**3.32**
TPS*Size	Phylo PCA	**1**	**0.051**	**0.10**	**1.64**	**1**	**0.052**	**0.10**	**1.77**
Residuals	Phylo PCA	13	0.199	0.40		13	0.177	0.36	
		Df	SS	R^2	Z	Df	SS	R^2	Z
Size	PACA	1	0.010	0.02	0.06	1	0.011	0.02	0.19
TPS	PACA	**1**	**0.190**	**0.43**	**2.90**	**1**	**0.199**	**0.45**	**3.07**
TPS*Size	PACA	**1**	**0.045**	**0.10**	**1.57**	**1**	**0.049**	**0.11**	**1.74**
Residuals	PACA	13	0.176	0.40		13	0.163	0.37	

Supplementary table 4: Estimated evolution rate matrix of the best model for specialist and generalist hunter in 90 % Phylogenetic PCs in “All taxa clade” analysis.

			Specialist		
	Comp1	Comp2	Comp3	Comp4	Comp5
Comp1	6.06E-04	-4.10E-04	1.89E-05	-6.10E-05	1.87E-04
Comp2	-4.10E-04	4.61E-04	-6.65E-05	-3.68E-05	-2.39E-04
Comp3	1.89E-05	-6.65E-05	4.33E-05	2.56E-05	7.92E-05
Comp4	-6.10E-05	-3.68E-05	2.56E-05	6.69E-05	4.14E-05
			Generalist		
	Comp1	Comp2	Comp3	Comp4	Comp5
Comp1	7.93E-04	-2.03E-05	1.60E-04	5.43E-05	9.27E-05
Comp2	-2.03E-05	4.10E-05	1.16E-05	-2.01E-05	-2.02E-06
Comp3	1.60E-04	1.16E-05	6.69E-05	4.79E-06	1.87E-05
Comp4	5.43E-05	-2.01E-05	4.79E-06	2.38E-05	-1.09E-05

Supplementary table 5: Estimated evolution rate matrix of the best model for specialist and generalist hunter in 90% Phylogenetic PCs in “Dilophosaurus clade” analysis.

		Specialist		
	Comp1	Comp2	Comp3	Comp4
Comp1	6.68E-04	5.65E-05	7.58E-05	5.08E-06
Comp2	5.65E-05	3.37E-04	-4.12E-05	-2.71E-06
Comp3	7.58E-05	-4.12E-05	5.12E-05	-4.13E-06
Comp4	5.08E-06	-2.71E-06	-4.13E-06	2.56E-05
		Generalist		
	Comp1	Comp2	Comp3	Comp4
Comp1	1.90E-04	-1.16E-04	-1.13E-04	-7.32E-06
Comp2	-1.16E-04	8.23E-05	7.39E-05	6.80E-06
Comp3	-1.13E-04	7.39E-05	7.56E-05	7.69E-06
Comp4	-7.32E-06	6.80E-06	7.69E-06	2.86E-05

Supplementary table 6: Estimated evolution rate matrix of the best model in removed-estimated taxa analysis for specialist and generalist hunter in 90 % Phylogenetic PCs in “All taxa clade”.

		Specialist		
	Comp1	Comp2	Comp3	Comp4
Comp1	4.47E-04	-7.62E-05	-7.51E-05	2.58E-06
Comp2	-7.62E-05	3.33E-04	-3.68E-05	-2.95E-06
Comp3	-7.51E-05	-3.68E-05	3.24E-05	-5.22E-06
Comp4	2.58E-06	-2.95E-06	-5.22E-06	2.71E-05
		Generalist		
	Comp1	Comp2	Comp3	Comp4
Comp1	2.83E-04	8.39E-05	1.11E-04	8.05E-07
Comp2	8.39E-05	3.92E-05	4.21E-05	6.44E-06
Comp3	1.11E-04	4.21E-05	5.32E-05	-6.01E-07
Comp4	8.05E-07	6.44E-06	-6.01E-07	2.30E-05

Supplementary table 7: Estimated evolution rate matrix of the best model in removed-estimated taxa analysis for specialist and generalist hunter in 90% Phylogenetic PCs in “Dilophosarus clade”.

		Specialist		
	Comp1	Comp2	Comp3	Comp4
Comp1	5.06E-04	-9.75E-05	5.40E-05	-1.95E-06
Comp2	-9.75E-05	3.58E-04	1.70E-05	-1.62E-05
Comp3	5.40E-05	1.70E-05	1.74E-05	2.59E-06
Comp4	-1.95E-06	-1.62E-05	2.59E-06	2.50E-05
		Generalist		
	Comp1	Comp2	Comp3	Comp4
Comp1	1.65E-04	7.01E-05	-1.02E-04	1.25E-05
Comp2	7.01E-05	4.01E-05	-5.05E-05	8.43E-06
Comp3	-1.02E-04	-5.05E-05	7.31E-05	-2.95E-06
Comp4	1.25E-05	8.43E-06	-2.95E-06	3.62E-05

Supplementary table 8: Confidence interval (IC) of 95% for the diagonal values of the evolution rate matrix in the best model for 90% of Phylo PCs in "All taxa clade" and "Dilophosaurus clade". * Show that the value of the parameter is outside of the Confidence interval (IC).

	Phylo-PCs	Lower IC	Upper IC	Observed		Lower IC	Upper IC	Observed
All taxa clade	PC1_ESP	0.00020	0.00112	0.00061	Dilophosaurus clade	0.00021	0.00128	0.00067
	PC2_ESP	0.00014	0.00091	0.00046		0.00012	0.00082	0.00034
	PC3_ESP	0.00001	0.00010	0.00004		0.00002	0.00012	0.00005
	PC4_ESP	0.00002	0.00017	0.00007		0.00001	0.00007	0.00003
	PC1_GEN	0.00025	0.00138	0.00079		0.00004	0.00062	0.00019
	PC2_GEN	0.00001	0.00015	0.00004		0.00001	0.00026	0.00008
	PC3_GEN	0.00002	0.00012	0.00007		0.00002	0.00017	0.00008
	PC4_GEN	0.00001	0.00005	0.00002		0.00001	0.00009	0.00003

Supplementary table 9: Confidence interval (IC) of 95% for the diagonal values of the evolution rate matrix in the best model for 90% of Phylo PCs in estimated taxa-removal analysis for "All taxa clade" and "Dilophosaurus clade". * Show that the value of the parameter is outside of the Confidence interval (IC).

	Phylo-PCs	Lower IC	Upper IC	Observed		Lower IC	Upper IC	Observed
All taxa clade	PC1_ESP	0.00011	0.00087	0.00045	Dilophosaurus clade	0.00013	0.00098	0.00051
	PC2_ESP	0.00008	0.00066	0.00033		0.00009	0.00073	0.00036
	PC3_ESP	0.00001	0.00007	0.00003		0.00001	0.00005	0.00002
	PC4_ESP	0.00001	0.00010	0.00003		0.00001	0.00009	0.00002
	PC1_GEN	0.00007	0.00055	0.00028		0.00004	0.00044	0.00016
	PC2_GEN	0.00001	0.00013	0.00004		0.00001	0.00020	0.00004
	PC3_GEN	0.00002	0.00012	0.00005		0.00002	0.00018	0.00007
	PC4_GEN	0.00001	0.00006	0.00002		0.00001	0.00013	0.00004

Bibliography

1. Baiano, M. A. Osteología y relaciones filogenéticas de *Aucasaurus garridoi* Coria, Chiape y Dinguis (Dinosauria, Theropoda, Abelisauridae). Implicancias Sistemáticas y Paleobiogeográficas en la evolución de los Abelisauridos. (Universidad Nacional del Comahue Centro Regional Universitario Bariloche, Rio Negro-Bariloche, 2021).
2. Ray, E. J., Horner, J. R., Smith, P. M., Thomason, J. J. & Upchurch, P. Cranial design and function in a large theropod dinosaur. **409**, (2001).
3. Cerroni, M. A., Canale, J. I. & Novas, F. E. The skull of *Carnotaurus sastrei* Bonaparte 1985 revisited: insights from craniofacial bones, palate and lower jaw. *Historical Biology* **33**, 2444–2485 (2021).
4. Sampson, S. D. & Witmer, L. M. CRANIOFACIAL ANATOMY OF *MAJUNGASAURUS CRENATISSIMUS* (THEROPODA: ABELISAURIDAE) FROM THE LATE CRETACEOUS OF MADAGASCAR. *Journal of Vertebrate Paleontology* **27**, 32–104 (2007).
5. Marsh, A. D. & Rowe, T. B. A comprehensive anatomical and phylogenetic evaluation of *Dilophosaurus wetherilli* (Dinosauria, Theropoda) with descriptions of new specimens from the Kayenta Formation of northern Arizona. *J. Paleontol.* **94**, 1–103 (2020).
6. Sereno, P. C. & Novas, F. E. The skull and neck of the basal theropod *Herrerasaurus ischigualastensis*. *Journal of Vertebrate Paleontology* **13**, 451–476 (1994).
7. Delcourt, R. Ceratosaur palaeobiology: new insights on evolution and ecology of the southern rulers. *Sci Rep* **8**, 9730 (2018).
8. Sereno, P. C., Wilson, J. A. & Conrad, J. L. New dinosaurs link southern landmasses in the Mid–Cretaceous. *Proc. R. Soc. Lond. B* **271**, 1325–1330 (2004).
9. Canale, J. I., Scanferla, C. A., Agnolin, F. L. & Novas, F. E. New carnivorous dinosaur from the Late Cretaceous of NW Patagonia and the evolution of abelisaurid theropods. *Naturwissenschaften* **96**, 409–414 (2009).
10. Zaher, H., Pol, D., Navarro, B. A., Delcourt, R. & Carvalho, A. B. An Early Cretaceous theropod dinosaur from Brazil sheds light on the cranial evolution of the Abelisauridae. *Comptes Rendus Palevol* (2020) doi: 10.5852/cr-palevol2020v19a6.

11. Gianechini, F. A. *et al.* A new furileusaurian abelisaurid from La Invernada (Upper Cretaceous, Santonian, Bajo de la Carpa Formation), northern Patagonia, Argentina. *Journal of Vertebrate Paleontology* **40**, e1877151 (2020).

12. Rowe, T. A new species of the theropod dinosaur *Syntarsus* from the Early Jurassic Kayenta Formation of Arizona. *Journal of Vertebrate Paleontology* **9**, 125–136 (1989).

13. Xu, X. *et al.* A Jurassic ceratosaur from China helps clarify avian digital homologies. *Nature* **459**, 940–944 (2009).
