# Supplementary material for: Morphology of the maxilla informs about the type of predation strategy in the evolution of Abelisauridae (Dinosauria: Theropoda)
Source: Sci Rep. 2025 Mar 6;15:7857. doi: 10.1038/s41598-025-87289-w (PMC11885552; doi:10.1038/s41598-025-87289-w)
Supplement: Supplementary file 2 — Supplementary Material 2 [file 41598_2025_87289_MOESM2_ESM.pdf]

	FAD	LAD
Aucasaurus	83.5	70.6
Abelisaurus	83.5	70.6
Allosaurus	155.7	150.8
Carnotaurus	72.1	66
AbeliLaColonia	72.1	66
Ceratosaurus	155.7	150.8
Dilophosaurus	199.3	182.7
Herrerasaurus	231	227
Majungasaurus	72.1	66
Masiakasaurus	72.1	66
Rugops	99.6	93.5
Skorpiovenator	99.6	89.8
Spectrovenator	125	113
Kryptops	125	100.5
Ekrixinatosaurus	99.6	93.5
Llukalkan	83.6	83.3
Syntarsus	199.3	182.7
Limusaurus	163.5	155.3
Noasaurus	70.6	66
Berberosaurus	190.8	174.1
Genyodectes	113	100.5
Huinculsaurus	99.6	89.8
Vespersaurus	125	72.1
Velocisaurus	83.6	83.3
Elaphrosaurus	155.7	93.5
Eoabelisaurus	179.29	177.86
Rajasaurus	71.1	66
Indosaurus	72.1	66
Arcovenator	72.1	70.6
Ilokelesia	99.6	89.8
Viavenator	86.6	83.3
Niebla	83.5	66
MPM99	100.5	86.3
Xenotarsosaurus	100.5	86.3
Laevisuchus	72.1	66
MNNtig6	145	132.6
CCG20011	161	155.3
Afromimus	125	100.5
