# Supplementary material for: Morphology of the maxilla informs about the type of predation strategy in the evolution of Abelisauridae (Dinosauria: Theropoda)
Source: Sci Rep. 2025 Mar 6;15:7857. doi: 10.1038/s41598-025-87289-w (PMC11885552; doi:10.1038/s41598-025-87289-w)
Supplement: Supplementary file 3 — Supplementary Material 3 [file 41598_2025_87289_MOESM3_ESM.pdf]

```r
set.seed(2024) #For reproducibility
# Abelisaurid Maxilla evolution
#Packages
library(geomorph)
library(ape)
library(mvMORPH)
library(RRPP)
library(geiger)
library(phytools)
library(paleotree)
library(strap)
library(geoscale)
library(bayou)
library(Claddis)
library(dplyr)
library(dispRity)
library(ggplot2)
library(nlme)
library(paleoTS)
library(fBasics)
library(MuMIn)
library(MASS)
library(vegan)
library(ggpubr)
library(rgl)
library(geometry)
library(webshot2)
library(surface)
#Geometric-Morphometric data and phylogenetic tree----
setwd() #Entri you working directory
Abelis<-readland.tps(file = "Supplementary_data_2.tps",readcurves = T) #Lanmark data
Abelis<-estimate.missing(A=Abelis,method="TPS") #Estimate missing landmarks
# we prepared semi landmarks to slide
lineasemi1 <- c(3,7:10,4)
lineasemi2 <- c(2,11:15,6)
lineasemi3 <- c(4,1:19,5)
lineasemi<-cbind(lineasemi1,lineasemi2,lineasemi3)
curve1<-matrix(0, nrow=4, ncol=3)
for(i in 1:nrow(curve1)){curve1[i,]<-c(lineasemi[i],lineasemi[i+1],lineasemi[i+2])}
#Procustes analysis
Abelis_gpa<-gpagen(A=Abelis,curves = curve1)

#Species names of landmark data
species <- c("Abelisaurus","Allosaurus","Aucasaurus","Carnotaurus" ,"Ceratosaurus",
"Dilophosaurus","Ekrixinatosaurus" ,"Herrerasaurus","Limusaurus","Llukalkan" ,
"Majungasaurus", "Masiakasaurus","Noasaurus", "Skorpiovenator", "Spectrovenator",
"Rugops","Syntarsus")
 for (i in 1:dim(Abelis_gpa$coords)[3]) {
    dimnames(Abelis_gpa$coords)[[3]][i] <- species[i]
 }

#Calibrated tree
AbeliPhy<-read.tree(file = "abelis_fully_resolved_tree_cal3_calibration.tre")
#We drop de taxa that have not maxilla
windows()
plot(AbeliPhy)
pr.species<-c("Berberosaurus","Genyodectes",
                                                          "Eoabelisaurus","Rajasaurus",
                                                          "Elaphrosaurus",
                                                          "Indosaurus","Arcovenator",
                                                          "Ilokelesia","Viavenator",
                                                          "Niebla","Huinculsaurus",
                                                          "Vespersaurus","Velocisaurus",
                                                          "Laevisuchus","MNNtig6",
                                                          "CCG20011","Xenotarsosaurus",
                                                          "MPM99","AbeliLaColonia")
nodes<-sapply(pr.species,grep,x=AbeliPhy$tip.label)

AbeliPhy<-drop.tip(AbeliPhy,pr.species)

AbeliPhy$edge.length[AbeliPhy$edge.length == 0] <- 0.01
```

```
windows()
plotTree(AbeliPhy,fsize=0.6,lwd=1,
                                    ftype="i")
# Now we can conduct Phylogenetic comparative analysis

#Evolution model without hyphotesis(SURFACE)----
#All taxa clade
Abelis_Phy_Coords<-geomorph.data.frame(coords=Abelis_gpa$coords,size=Abelis_gpa$Csize,
Phy = AbeliPhy)
Abelis_PhyPCA<-gm.prcomp(A=Abelis_Phy_Coords$coords,phy =Abelis_Phy_Coords$Phy,GLS=
TRUE)
#Dilophosaurus clade
#pr.species<-c("Herrerasaurus","Syntarsus")
nodes<-sapply(pr.species,grep,x=AbeliPhy$tip.label)

AbeliPhy<-drop.tip(AbeliPhy,pr.species)

AbeliPhy$edge.length[AbeliPhy$edge.length == 0] <- 0.01
plot(AbeliPhy)

#procutes coordinates object
Abelis<-Abelis_gpa$coords[,,c("Abelisaurus","Aucasaurus","Carnotaurus",
"Ekrixinatosaurus","Majungasaurus","Skorpiovenator","Llukalkan","Spectrovenator",
"Rugops","Ceratosaurus","Noasaurus","Limusaurus","Masiakasaurus","Dilophosaurus",
"Allosaurus")]
#Phylogenetic Data frame
Abelis_Phy_Coords<-geomorph.data.frame(coords=Abelis,Phy = AbeliPhy)

Abelis_PhyPCA<-gm.prcomp(A=Abelis_Phy_Coords$coords,phy =Abelis_Phy_Coords$Phy,GLS=
TRUE)
#Removal estimated taxa analysis
pr.species<-c("Llukalkan","Abelisaurus")
nodes<-sapply(pr.species,grep,x=AbeliPhy$tip.label)

AbeliPhy<-drop.tip(AbeliPhy,pr.species)

AbeliPhy$edge.length[AbeliPhy$edge.length == 0] <- 0.01
plot(AbeliPhy)

#We make a new procutes coordinates object
##Without Herrerasaurus and Syntarsus
Abelis<-Abelis_gpa$coords[,,c("Aucasaurus","Carnotaurus","Ekrixinatosaurus",
"Majungasaurus","Skorpiovenator","Spectrovenator","Rugops","Ceratosaurus","Noasaurus",
"Limusaurus","Masiakasaurus","Dilophosaurus","Allosaurus")]
#With Herrerasaurus and Syntarsus
Abelis<-Abelis_gpa$coords[,,c("Aucasaurus","Carnotaurus","Ekrixinatosaurus",
"Majungasaurus","Skorpiovenator","Spectrovenator","Rugops","Ceratosaurus","Noasaurus",
"Limusaurus","Masiakasaurus","Dilophosaurus","Allosaurus","Herrerasaurus","Syntarsus"
)]

#Phylogenetic data frame
Abelis_Phy_Coords<-geomorph.data.frame(coords=Abelis,Phy = AbeliPhy)

#We have to calculate again the PhyloPCA
Abelis_PhyPCA<-gm.prcomp(A=Abelis_Phy_Coords$coords,phy =Abelis_Phy_Coords$Phy,GLS=
TRUE)

#Notice that first you have to choise one of the data sets above before you run
SURFACE analysis.
AbeliPhy<-nameNodes(AbeliPhy)
#Data transformation for SURFACE
data.dino<-data.frame(PC1_Phy=Abelis_PhyPCA$x[,1],PC2_Phy=Abelis_PhyPCA$x[,2], PC3_phy
=Abelis_PhyPCA$x[,3],PC4_Phy=Abelis_PhyPCA$x[,4])
olist<-convertTreeData(AbeliPhy,data.dino)
otree<-olist[[1]]
odata<-olist[[2]]
#We look for phenotipyc optima
x<-surfaceForward(otree,odata, aic_threshold = 0, exclude = 0)
surfaceSummary(x)
k<-length(x)
#Plot of how many optima SURFACE fou
```

```
windows()
surfaceTreePlot(AbeliPhy, x[[k]], labelshifts = T)
#Evolutionary trends, Phylogenetic signal and PGLS analysis----

#Ecological category whit Spectrovenator as Generalis sp (Prior Hypothesis)
eco<-c("Esp","Gen","Esp","Esp","Gen","Gen","Esp","Gen","Gen",
                            "Esp","Esp","Gen","Gen","Esp","Gen","Esp","Gen")
#Ecological category whit Spectrovenator as specialis sp (Posterior hypothesis)
ecoE<-c("Esp","Gen","Esp","Esp","Gen","Gen","Esp","Gen","Gen",
                            "Esp","Esp","Gen","Gen","Esp","Esp","Esp","Gen")
#Phylogenetic Data frame  (Prior Hypothesis)
#Notice that first we have to select what eco data we can run (change eco object in Ecologia groups)
Abelis_Phy_Coords<-geomorph.data.frame(coords=Abelis_gpa$coords,size=Abelis_gpa$Csize, Phy = AbeliPhy,Ecologia=eco,Especie=species)
#Phylogenetic Data frame whit (Posterior hypothesis)
Abelis_Phy_Coords<-geomorph.data.frame(coords=Abelis_gpa$coords,size=Abelis_gpa$Csize, Phy = AbeliPhy,Ecologia=ecoE,Especie=species)

#Phylo PCA
Abelis_PhyPCA<-gm.prcomp(A=Abelis_Phy_Coords$coords,phy =Abelis_Phy_Coords$Phy,GLS=TRUE)
summary(Abelis_PhyPCA)
#PACA
Abelis_PaCA<-gm.prcomp(A=Abelis_Phy_Coords$coords,phy =Abelis_Phy_Coords$Phy,GLS=TRUE, align.to.phy = TRUE,transform = FALSE)
#Levels of the factor to plot
gp <- as.factor(paste(Abelis_Phy_Coords$Ecologia))
levels(gp)
#Phylo PCA
windows()
plot(Abelis_PhyPCA, axis1=1, axis2= 2, phylo =TRUE,cex=2, pch= 19, col=gp,  phylo.par= list( tip.labels =TRUE, node.labels=FALSE, anc.states= FALSE, edge.color= "black", edge.width= 1, tip.txt.cex =0.5, tip.txt.adj = c(-0.1, -0.1) ))

#PACA
windows()
plot(Abelis_PaCA, axis1=1, axis2= 2, phylo =TRUE,cex=2, pch= 19,col=gp,  phylo.par= list( tip.labels =TRUE, node.labels=FALSE, anc.states= F, edge.color= "black", edge.width= 1, tip.txt.cex =0.5, tip.txt.adj = c(-0.1, -0.1) ))
dev.off()
physig.Abelis<-physignal.z(A= Abelis_Phy_Coords$coords,  phy= Abelis_Phy_Coords$Phy, iter = 100000)
summary(physig.Abelis)

#PGLS
#If we can run the PGLS analysis in Posterior hypothesis we have to change the Ecologia in the data frame
#Procustes coordinates
Phy_eco<-procD.pgls(Abelis_Phy_Coords$coords~Abelis_Phy_Coords$Ecologia*Abelis_Phy_Coords$size,phy = Abelis_Phy_Coords$Phy,SS.type = "II",iter = 999)
summary(Phy_eco)
#PGLS in Phylo PCA
Phy_eco<-procD.pgls(Abelis_PhyPCA$x[,1:4]~Abelis_Phy_Coords$Ecologia*Abelis_Phy_Coords$size,phy = Abelis_Phy_Coords$Phy,SS.type = "II",iter = 999)
summary(Phy_eco)
#PGLS in PACA
Phy_eco<-procD.pgls(Abelis_PaCA$x[,1:4]~Abelis_Phy_Coords$Ecologia*Abelis_Phy_Coords$size,phy = Abelis_Phy_Coords$Phy,SS.type = "II",iter = 999)
summary(Phy_eco)

#Phylogenetic Signal
#Procustes coordinates
physig.Abelis<-physignal.z(A= Abelis_Phy_Coords$coords, phy= Abelis_Phy_Coords$Phy, iter = 100000)
summary(physig.Abelis)
#PhyloPCA
physig.Abelis<-physignal.z(A= Abelis_PhyPCA$x[,1:3], phy= Abelis_Phy_Coords$Phy, iter = 100000)
#PACA
physig.Abelis<-physignal.z(A= Abelis_PaCA$x[,1:5], phy= Abelis_Phy_Coords$Phy, iter =
```

```r
100000)


#Disparity trough time analysis----
windows()
x<-dtt(AbeliPhy, Abelis_PhyPCA$x[,1:4], index="avg.sq",
                                mdi.range=c(0,1), nsim=10000, CI=0.95, plot=TRUE, calculateMDIp=T)
#Evolutions models, comparing the Prior and Posterior hypothesis in all taxa clade ----
#We mapped the ecological character in the tree with Spectrovenator as a Gen (Prior hypothesis)
state<-as.vector(c(rep("ESP",8),rep("GEN",9)));names(state)<-AbeliPhy$tip.label
#We mapped the ecological character in the tree with Spectrovenator as a Esp (Posterior Hypothesis)
state1<-as.vector(c(rep("ESP",9),rep("GEN",8)));names(state1)<-AbeliPhy$tip.label
#We create the Prior Hyphotesis
treeAbeli<-make.simmap(AbeliPhy, state, model="ER", nsim=1)

plot(treeAbeli)
#We create the Posterior Hyphotesis obtained by SURFACE
treeAbeli1<-make.simmap(AbeliPhy, state1, model="ER", nsim=1)
plot(treeAbeli1)

#The first four components of Phylo PCA explained the 90% of variance in data
OU1<-mvOU(treeAbeli, Abelis_PhyPCA$x[,1:4], model="OU1")
OUM<-mvOU(treeAbeli, Abelis_PhyPCA$x[,1:4], model="OUM") #Prior hypohtesis
BM1<-mvBM(treeAbeli, Abelis_PhyPCA$x[,1:4], model="BM1")
BMM<-mvBM(treeAbeli, Abelis_PhyPCA$x[,1:4], model="BMM")#Prior hypohtesis
EB<-mvEB(treeAbeli, Abelis_PhyPCA$x[,1:4], diagnostic=FALSE, echo=FALSE)
BMMS<-mvBM(treeAbeli1, Abelis_PhyPCA$x[,1:4], model="BMM")#Posterior hypohtesis
OUMS<-mvOU(treeAbeli1, Abelis_PhyPCA$x[,1:4], model="OUM")#Posterior hypohtesis

#AKAIKE criterion information, weight and Looklihood
AIC<-data.frame(AIC=c(OU1$AIC,OUM$AIC,BM1$AIC,BMM$AIC,EB$AIC,BMMS$AIC,OUMS$AIC))
LogLike<-data.frame(LogLik=c(OU1$LogLik,OUM$LogLik,BM1$LogLik,BMM$LogLik,EB$LogLik,BMMS$LogLik,OUMS$LogLik))
results<-c(OU1$AIC,OUM$AIC,BM1$AIC,BMM$AIC,EB$AIC,BMMS$AIC,OUMS$AIC)
aicw(results)

#Evolutions models, comparing the Prior and Posterior hypothesis in Dilophosaurus clade ----
#We drop Herrerasaurus and Syntarsus
pr.species<-c("Herrerasaurus","Syntarsus")
nodes<-sapply(pr.species,grep,x=AbeliPhy$tip.label)

AbeliPhy<-drop.tip(AbeliPhy,pr.species)

AbeliPhy$edge.length[AbeliPhy$edge.length == 0] <- 0.01
plot(AbeliPhy)

#We make a new procutes coordinates object
Abelis<-Abelis_gpa$coords[,,c("Abelisaurus","Aucasaurus","Carnotaurus","Ekrixinatosaurus","Majungasaurus","Skorpiovenator","Llukalkan","Spectrovenator","Rugops","Ceratosaurus","Noasaurus","Limusaurus","Masiakasaurus","Dilophosaurus","Allosaurus")]
#Phylogenetic Data frame
Abelis_Phy_Coords<-geomorph.data.frame(coords=Abelis,Phy = AbeliPhy)

#We have to perform a new Phylo PCA
Abelis_PhyPCA<-gm.prcomp(A=Abelis_Phy_Coords$coords,phy =Abelis_Phy_Coords$Phy,GLS=TRUE)

#We mapped the ecological character in the tree with Spectrovenator as a Gen (Prior Hypothesis)
state<-as.vector(c(rep("ESP",8),rep("GEN",7)));names(state)<-AbeliPhy$tip.label
#We mapped the ecological character in the tree with Spectrovenator as a Esp (Posterior Hypothesis)
state1<-as.vector(c(rep("ESP",9),rep("GEN",6)));names(state1)<-AbeliPhy$tip.label
#We create the Prior Hypothesis
treeAbeli<-make.simmap(AbeliPhy, state, model="ER", nsim=1)
```

```
plot(treeAbeli)
#We create the Posterior Hypothesis
treeAbeli1<-make.simmap(AbeliPhy, state1, model="ER", nsim=1)
plot(treeAbeli1)
#The first two components
OU1<-mvOU(treeAbeli, Abelis_PhyPCA$x[,1:4], model="OU1")
#Prior Hypothesis
OUM<-mvOU(treeAbeli, Abelis_PhyPCA$x[,1:4], model="OUM")
BM1<-mvBM(treeAbeli, Abelis_PhyPCA$x[,1:4], model="BM1")
#Prior Hypothesis
BMM<-mvBM(treeAbeli, Abelis_PhyPCA$x[,1:4], model="BMM")
EB<-mvEB(treeAbeli, Abelis_PhyPCA$x[,1:4], diagnostic=FALSE, echo=FALSE)
#Posterior Hypothesis
BMMS<-mvBM(treeAbeli1, Abelis_PhyPCA$x[,1:4], model="BMM")
#Posterior Hypothesis
OUMS<-mvOU(treeAbeli1, Abelis_PhyPCA$x[,1:4], model="OUM")

#AKAIKE criterion information, weight and Looklihood
AIC<-data.frame(AIC=c(OU1$AIC,OUM$AIC,BM1$AIC,BMM$AIC,EB$AIC,BMMS$AIC,OUMS$AIC))
LogLike<-data.frame(LogLik=c(OU1$LogLik,OUM$LogLik,BM1$LogLik,BMM$LogLik,EB$LogLik,BMMS$LogLik,OUMS$LogLik))
results<-c(OU1$AIC,OUM$AIC,BM1$AIC,BMM$AIC,EB$AIC,BMMS$AIC,OUMS$AIC)
aicw(results)

#removal estimated taxa analysis----
#Notice that if you want to run this section in all taxa clade you will need re run the all taxa clade
pr.species<-c("Llukalkan","Abelisaurus")
nodes<-sapply(pr.species,grep,x=AbeliPhy$tip.label)

AbeliPhy<-drop.tip(AbeliPhy,pr.species)

AbeliPhy$edge.length[AbeliPhy$edge.length == 0] <- 0.01
plot(AbeliPhy)

#We make a new procutes coordinates object
##Without Herrerasaurus and Syntarsus
Abelis<-Abelis_gpa$coords[,,c("Aucasaurus","Carnotaurus","Ekrixinatosaurus","Majungasaurus","Skorpiovenator","Spectrovenator","Rugops","Ceratosaurus","Noasaurus","Limusaurus","Masiakasaurus","Dilophosaurus","Allosaurus")]
#With Herrerasaurus and Syntarsus
Abelis<-Abelis_gpa$coords[,,c("Aucasaurus","Carnotaurus","Ekrixinatosaurus","Majungasaurus","Skorpiovenator","Spectrovenator","Rugops","Ceratosaurus","Noasaurus","Limusaurus","Masiakasaurus","Dilophosaurus","Allosaurus","Herrerasaurus","Syntarsus")]

#Phylogenetic data frame
Abelis_Phy_Coords<-geomorph.data.frame(coords=Abelis,Phy = AbeliPhy)

#We have to calculate again the PhyloPCA
Abelis_PhyPCA<-gm.prcomp(A=Abelis_Phy_Coords$coords,phy =Abelis_Phy_Coords$Phy,GLS=TRUE)

#We mapped the ecological character in the tree with Spectrovenator as a Gen (Prior Hyphotesis)
state<-as.vector(c(rep("ESP",6),rep("GEN",9)));names(state)<-AbeliPhy$tip.label
#We mapped the ecological character in the tree with Spectrovenator as a Esp (Prior Hypothesis)
state1<-as.vector(c(rep("ESP",7),rep("GEN",8)));names(state1)<-AbeliPhy$tip.label
#We create the Prior ypothesis
treeAbeli<-make.simmap(AbeliPhy, state, model="ER", nsim=1)

plot(treeAbeli)
#We create the posterior hypothesis
treeAbeli1<-make.simmap(AbeliPhy, state1, model="ER", nsim=1)
plot(treeAbeli1)
#Evolutions models
#The first two components
OU1<-mvOU(treeAbeli, Abelis_PhyPCA$x[,1:4], model="OU1")
#Prior hypothesis
```

```
OUM<-mvOU(treeAbeli, Abelis_PhyPCA$x[,1:4], model="OUM")
BM1<-mvBM(treeAbeli, Abelis_PhyPCA$x[,1:4], model="BM1")
#Prior Hypothesis
BMM<-mvBM(treeAbeli, Abelis_PhyPCA$x[,1:4], model="BMM")
EB<-mvEB(treeAbeli, Abelis_PhyPCA$x[,1:4], diagnostic=FALSE, echo=FALSE)
#Posterior Hypothesis
BMMS<-mvBM(treeAbeli1, Abelis_PhyPCA$x[,1:4], model="BMM")
#Posterior Hypothesis
OUMS<-mvOU(treeAbeli1, Abelis_PhyPCA$x[,1:4], model="OUM")
#AKAIKE criterion information, weight and Looklihood
AIC<-data.frame(AIC=c(OU1$AIC,OUM$AIC,BM1$AIC,BMM$AIC,EB$AIC,BMMS$AIC,OUMS$AIC))
LogLike<-data.frame(LogLik=c(OU1$LogLik,OUM$LogLik,BM1$LogLik,BMM$LogLik,EB$LogLik,
BMMS$LogLik,OUMS$LogLik))
results<-c(OU1$AIC,OUM$AIC,BM1$AIC,BMM$AIC,EB$AIC,BMMS$AIC,OUMS$AIC)
aicw(results)
#Morphological changes----
#Links between landmarks
s<-c(1,16,17,18,5,5,6,14,13,12,11,2,2,3,7,8,9,4)
d<-c(16,17,18,19,19,6,15,15,14,13,12,11,3,7,8,9,10,10)
x<-cbind(s,d)
PCAMAX1<-PCA$shapes$shapes.comp1$max
PCAMIN1<-PCA$shapes$shapes.comp1$min
PCAMAX2<-PCA$shapes$shapes.comp2$max
PCAMIN2<-PCA$shapes$shapes.comp2$min
PCAMEAN<-mshape(PCA$shapes$shapes.comp1)

#Mean shape
plotRefToTarget(M1=PCAMEAN,M2=PCAMEAN,method = "TPS",links = z)
dev.off()
#Phylo PC 1 maximum shape
plotRefToTarget(M1=PCAMAX1,M2=PCAMEAN,method = "TPS",links = x)

#Phylo PC 1 minimum shape
plotRefToTarget(M1=Abelis_PhyPCA$shapes$shapes.comp2$min,M2=mshape(Abelis_PhyPCA$
shapes$shapes.comp1),method = "TPS",links = x)
#Phylo PC 2 maximum shape
plotRefToTarget(M1=PCAMAX2,M2=PCAMEAN,method = "TPS",links = x)
#Phylo PC 2 minimu shape
plotRefToTarget(M1=PCAMIN2,M2=PCAMEAN,method = "TPS",links = x)
```
