# Supplementary material for: Morphology of the maxilla informs about the type of predation strategy in the evolution of Abelisauridae (Dinosauria: Theropoda)
Source: Sci Rep. 2025 Mar 6;15:7857. doi: 10.1038/s41598-025-87289-w (PMC11885552; doi:10.1038/s41598-025-87289-w)
Supplement: Supplementary file 4 — Supplementary Material 4 [file 41598_2025_87289_MOESM4_ESM.pdf]

ne/ Quilmesaurus Vespersaurus Velocisaurus Kurupi Elemgasem Rahiolisaurus Pycnonemosaurus Genusaurus Dahalokely Kryptops;
