# Supplementary material for: Morphology of the maxilla informs about the type of predation strategy in the evolution of Abelisauridae (Dinosauria: Theropoda)
Source: Sci Rep. 2025 Mar 6;15:7857. doi: 10.1038/s41598-025-87289-w (PMC11885552; doi:10.1038/s41598-025-87289-w)
Supplement: Supplementary file 6 — Supplementary Material 6 [file 41598_2025_87289_MOESM6_ESM.pdf]

```r
set.seed(2024) #For reproducibility
# Abelisaurid Maxilla evolution

#Packages
library(geomorph)
library(ape)
library(mvMORPH)
library(RRPP)
library(geiger)
library(phytools)
library(paleotree)
library(strap)
library(geoscale)
library(bayou)
library(Claddis)
library(dplyr)
library(dispRity)
library(ggplot2)
library(nlme)
library(paleoTS)
library(fBasics)
library(MuMIn)
library(MASS)
library(vegan)
library(ggpubr)
library(rgl)
library(geometry)
library(webshot2)
library(surface)
#Notice that before to run simulation analysis you will need to run first the objects (Geometric morphometric data, Phylogenetyc tree and Phylo PCA) in an especific data sets ("All taxa clade", "Dilophosaurus clade" and removed estimated taxa analysis) in Macroevolutionary analysis to then build the interval confidence.
#The BMM model was always the best model, but was different between Hypothesis in data sets, so that you must change the treeabeli (Prior hypothesis) for treeabeli1 (Posterior hypothesis) phylogenetic tree if you want to build the confidence interval.


BMM90<-mvBM(treeAbeli1, Abelis_PhyPCA$x[,1:4], model="BMM")
simul4<-mvSIM(treeAbeli,nsim=500,param = BMM90)
#Fitting the best model to all data set simulated
results<-lapply(simul4,function(x){
    mvBM(treeAbeli,x,model="BMM",method="rpf", echo=FALSE,diagnostic=FALSE)
})
sigma_simul_Esp_PC1<-sapply(results,function(x){x$sigma[1,1,"ESP"]})
sigma_simul_Esp_PC2<-sapply(results,function(x){x$sigma[2,2,"ESP"]})
sigma_simul_Esp_PC3<-sapply(results,function(x){x$sigma[3,3,"ESP"]})
sigma_simul_Esp_PC4<-sapply(results,function(x){x$sigma[4,4,"ESP"]})


sigma_simul_Gen_PC1<-sapply(results,function(x){x$sigma[1,1,"GEN"]})
sigma_simul_Gen_PC2<-sapply(results,function(x){x$sigma[2,2,"GEN"]})
sigma_simul_Gen_PC3<-sapply(results,function(x){x$sigma[3,3,"GEN"]})
sigma_simul_Gen_PC4<-sapply(results,function(x){x$sigma[4,4,"GEN"]})

#Create a confidence interval
##PhyloPC1 for specialist hunters
ICEsp1<-quantile(sigma_simul_Esp_PC1,c(0.025,0.975))
#PhyloPC2 for specialist hunters
ICEsp2<-quantile(sigma_simul_Esp_PC2,c(0.025,0.975))
#PhyloPC3 for specialist hunters
ICEsp3<-quantile(sigma_simul_Esp_PC3,c(0.025,0.975))
#PhyloPC4 for specialist hunters
ICEsp4<-quantile(sigma_simul_Esp_PC4,c(0.025,0.975))
#PhyloPC1 for generalist hunters
ICGen1<-quantile(sigma_simul_Gen_PC1,c(0.025,0.975))
#PhyloPC2 for generalist hunters
ICGen2<-quantile(sigma_simul_Gen_PC2,c(0.025,0.975))
#PhyloPC3 for generalist hunters
ICGen3<-quantile(sigma_simul_Gen_PC3,c(0.025,0.975))
```

```
#PhyloPC4 for generalist hunters
ICGen4<-quantile(sigma_simul_Gen_PC4,c(0.025,0.975))
```
