# Supplementary material for: Morphology of the maxilla informs about the type of predation strategy in the evolution of Abelisauridae (Dinosauria: Theropoda)
Source: Sci Rep. 2025 Mar 6;15:7857. doi: 10.1038/s41598-025-87289-w (PMC11885552; doi:10.1038/s41598-025-87289-w)
Supplement: Supplementary file 7 — Supplementary Material 7 [file 41598_2025_87289_MOESM7_ESM.pdf]

```
LM=6
262.00000 614.00000
261.00000 515.00000
149.00000 537.00000
192.00000 614.00000
NA NA
NA NA
CURVES=3
POINTS=4
152.00000 544.00000
158.00000 566.00000
170.00000 587.00000
185.00000 605.00000
POINTS=5
NA NA
NA NA
NA NA
NA NA
NA NA
POINTS=4
NA NA
NA NA
NA NA
NA NA
IMAGE=Abelisaurus.jpg
ID=Abelisaurus
SCALE=0.009344
LM=6
236.00000 397.00000
240.00000 357.00000
165.00000 356.00000
175.00000 395.00000
349.00000 377.00000
388.00000 368.00000
CURVES=3
POINTS=4
164.00000 364.00000
165.00000 374.00000
168.00000 383.00000
176.00000 390.00000
POINTS=5
255.00000 358.00000
284.00000 357.00000
312.00000 361.00000
340.00000 363.00000
369.00000 365.00000
POINTS=4
247.00000 385.00000
277.00000 378.00000
307.00000 374.00000
338.00000 375.00000
IMAGE=Allosaurus.jpg
ID=Allosaurus
SCALE=0.169273
LM=6
1740.00000 1954.00000
1700.00000 1005.00000
923.00000 1124.00000
1134.00000 1940.00000
3399.00000 1744.00000
3794.00000 1278.00000
CURVES=3
POINTS=4
920.00000 1174.00000
938.00000 1426.00000
1009.00000 1668.00000
1106.00000 1901.00000
POINTS=5
1801.00000 983.00000
2244.00000 1032.00000
2680.00000 1118.00000
```

```
3110.00000 1230.00000
3553.00000 1282.00000
POINTS=4
1797.00000 1881.00000
2228.00000 1778.00000
2630.00000 1785.00000
3201.00000 1755.00000
IMAGE=Aucasaurus.jpg
ID=Aucasaurus
SCALE=0.023696
LM=6
141.00000 241.00000
133.00000 95.00000
34.00000 122.00000
71.00000 241.00000
252.00000 178.00000
385.00000 83.00000
CURVES=3
POINTS=4
33.00000 142.00000
40.00000 174.00000
56.00000 202.00000
68.00000 232.00000
POINTS=5
155.00000 87.00000
208.00000 84.00000
261.00000 85.00000
313.00000 89.00000
366.00000 86.00000
POINTS=4
151.00000 228.00000
176.00000 211.00000
205.00000 201.00000
235.00000 195.00000
IMAGE=Carnotaurus.png
ID=Carnotaurus
SCALE=0.074066
LM=6
91.00000 110.00000
91.00000 84.00000
54.00000 90.00000
55.00000 110.00000
165.00000 110.00000
192.00000 101.00000
CURVES=3
POINTS=4
54.00000 94.00000
54.00000 98.00000
54.00000 102.00000
55.00000 106.00000
POINTS=5
106.00000 82.00000
124.00000 81.00000
142.00000 85.00000
160.00000 90.00000
178.00000 94.00000
POINTS=4
101.00000 103.00000
118.00000 100.00000
133.00000 100.00000
152.00000 104.00000
IMAGE=Ceratosaurus.jpg
ID=Ceratosaurus
SCALE=0.256074
LM=6
223.00000 246.00000
220.00000 190.00000
133.00000 212.00000
150.00000 246.00000
388.00000 241.00000
448.00000 193.00000
```

```
CURVES=3
POINTS=4
133.00000 217.00000
133.00000 225.00000
136.00000 233.00000
142.00000 239.00000
POINTS=5
243.00000 190.00000
288.00000 193.00000
334.00000 198.00000
379.00000 203.00000
424.00000 199.00000
POINTS=4
236.00000 233.00000
274.00000 229.00000
326.00000 227.00000
359.00000 235.00000
IMAGE=Dilophosaurus.jpg
ID=Dilophosaurus
SCALE=0.056176
LM=6
219.00000 338.00000
227.00000 106.00000
114.00000 114.00000
135.00000 336.00000
454.00000 298.00000
727.00000 214.00000
CURVES=3
POINTS=4
102.00000 135.00000
98.00000 196.00000
105.00000 258.00000
124.00000 316.00000
POINTS=5
270.00000 107.00000
378.00000 113.00000
485.00000 127.00000
591.00000 151.00000
694.00000 184.00000
POINTS=4
232.00000 320.00000
274.00000 302.00000
362.00000 292.00000
422.00000 300.00000
IMAGE=Ekrixinatosaurus.jpg
ID=Ekrixinatosaurus
SCALE=0.056815
LM=6
256.00000 577.00000
256.00000 521.00000
150.00000 532.00000
177.00000 576.00000
355.00000 551.00000
427.00000 526.00000
CURVES=3
POINTS=4
154.00000 543.00000
157.00000 554.00000
164.00000 563.00000
172.00000 571.00000
POINTS=5
274.00000 521.00000
307.00000 523.00000
340.00000 526.00000
373.00000 528.00000
406.00000 527.00000
POINTS=4
261.00000 568.00000
285.00000 559.00000
315.00000 555.00000
344.00000 553.00000
```

```
IMAGE=Herrerasaurus.jpg
ID=Herrerasaurus
SCALE=0.113519
LM=6
81.00000 78.00000
81.00000 68.00000
67.00000 69.00000
74.00000 78.00000
160.00000 66.00000
170.00000 61.00000
CURVES=3
POINTS=4
68.00000 71.00000
69.00000 73.00000
70.00000 74.00000
71.00000 75.00000
POINTS=5
92.00000 68.00000
109.00000 67.00000
126.00000 65.00000
142.00000 64.00000
159.00000 62.00000
POINTS=4
84.00000 75.00000
106.00000 72.00000
127.00000 70.00000
149.00000 67.00000
IMAGE=Limusaurus.jpg
ID=Limusaurus
SCALE=0.032241
LM=6
233.00000 795.00000
238.00000 672.00000
116.00000 677.00000
131.00000 793.00000
423.00000 767.00000
536.00000 667.00000
CURVES=3
POINTS=4
115.00000 694.00000
117.00000 721.00000
120.00000 748.00000
125.00000 775.00000
POINTS=5
266.00000 670.00000
326.00000 672.00000
385.00000 678.00000
445.00000 669.00000
505.00000 668.00000
POINTS=4
243.00000 785.00000
277.00000 772.00000
358.00000 758.00000
408.00000 761.00000
IMAGE=Llukalcan.jpg
ID=Llukalcan
SCALE=0.049990
LM=6
155.00000 199.00000
161.00000 48.00000
20.00000 94.00000
65.00000 199.00000
291.00000 141.00000
421.00000 47.00000
CURVES=3
POINTS=4
19.00000 106.00000
22.00000 137.00000
38.00000 165.00000
54.00000 192.00000
POINTS=5
```

```
182.00000 48.00000
235.00000 48.00000
288.00000 51.00000
341.00000 52.00000
394.00000 48.00000
POINTS=4
159.00000 185.00000
186.00000 173.00000
227.00000 162.00000
275.00000 147.00000
IMAGE=Majungasaurus_left.png
ID=Majungasaurus
SCALE=0.071421
LM=6
103.00000 166.00000
102.00000 147.00000
73.00000 154.00000
84.00000 167.00000
191.00000 131.00000
201.00000 122.00000
CURVES=3
POINTS=4
75.00000 156.00000
77.00000 159.00000
79.00000 161.00000
81.00000 163.00000
POINTS=5
115.00000 143.00000
134.00000 139.00000
152.00000 135.00000
170.00000 129.00000
189.00000 125.00000
POINTS=4
105.00000 160.00000
120.00000 152.00000
152.00000 142.00000
180.00000 134.00000
IMAGE=Masiakasaurus.jpg
ID=Masiakasaurus
SCALE=0.043853
LM=6
996.00000 1086.00000
996.00000 866.00000
625.00000 863.00000
773.00000 1104.00000
1596.00000 946.00000
1782.00000 801.00000
CURVES=3
POINTS=4
625.00000 896.00000
656.00000 964.00000
706.00000 1021.00000
756.00000 1077.00000
POINTS=5
1078.00000 856.00000
1254.00000 852.00000
1402.00000 850.00000
1562.00000 825.00000
1719.00000 805.00000
POINTS=4
1028.00000 1058.00000
1202.00000 1024.00000
1412.00000 993.00000
1603.00000 955.00000
IMAGE=Noasaurus.jpg
ID=Noasaurus
SCALE=0.006968
LM=6
157.00000 204.00000
158.00000 118.00000
90.00000 151.00000
```

```
99.00000 205.00000
209.00000 187.00000
295.00000 105.00000
CURVES=3
POINTS=4
91.00000 160.00000
91.00000 173.00000
93.00000 186.00000
96.00000 197.00000
POINTS=5
174.00000 114.00000
201.00000 111.00000
228.00000 108.00000
255.00000 109.00000
282.00000 104.00000
POINTS=4
162.00000 198.00000
173.00000 190.00000
188.00000 189.00000
203.00000 187.00000
IMAGE=Skorpiovenator.jpg
ID=Skorpiovenator
SCALE=0.079206
LM=6
292.00000 513.00000
290.00000 398.00000
175.00000 430.00000
233.00000 515.00000
474.00000 480.00000
583.00000 424.00000
CURVES=3
POINTS=4
181.00000 444.00000
194.00000 463.00000
205.00000 482.00000
221.00000 502.00000
POINTS=5
316.00000 393.00000
374.00000 395.00000
430.00000 408.00000
487.00000 421.00000
545.00000 428.00000
POINTS=4
300.00000 503.00000
330.00000 489.00000
381.00000 480.00000
454.00000 479.00000
IMAGE=Spectrovenator.jpg
ID=Spectrovenator
SCALE=0.029062
LM=6
91.00000 81.00000
89.00000 32.00000
41.00000 49.00000
55.00000 81.00000
151.00000 63.00000
191.00000 39.00000
CURVES=3
POINTS=4
39.00000 54.00000
40.00000 63.00000
44.00000 71.00000
50.00000 77.00000
POINTS=5
102.00000 28.00000
122.00000 28.00000
142.00000 29.00000
162.00000 29.00000
182.00000 33.00000
POINTS=4
97.00000 75.00000
```

```
109.00000 69.00000
124.00000 63.00000
141.00000 63.00000
IMAGE=Rugops.jpg
ID=Rugops
SCALE=0.172388
LM=6
147.00000 183.00000
144.00000 167.00000
117.00000 171.00000
127.00000 185.00000
294.00000 173.00000
313.00000 165.00000
CURVES=3
POINTS=4
116.00000 176.00000
117.00000 179.00000
119.00000 182.00000
123.00000 183.00000
POINTS=5
164.00000 165.00000
197.00000 166.00000
230.00000 165.00000
264.00000 164.00000
297.00000 163.00000
POINTS=4
153.00000 178.00000
176.00000 177.00000
211.00000 177.00000
280.00000 173.00000
IMAGE=Syntarsus.png
ID=Syntarsus
SCALE=0.072395
```
