# Supplementary material for: Morphology of the maxilla informs about the type of predation strategy in the evolution of Abelisauridae (Dinosauria: Theropoda)
Source: Sci Rep. 2025 Mar 6;15:7857. doi: 10.1038/s41598-025-87289-w (PMC11885552; doi:10.1038/s41598-025-87289-w)
Supplement: Supplementary file 8 — Supplementary Material 8 [file 41598_2025_87289_MOESM8_ESM.pdf]

```
set.seed(2024)

library("Claddis")
library("dplyr")
library("dispRity")
library("ggplot2")
library("nlme")
library("ape")
library("paleotree")
library("paleoTS")
library("phytools")
library("strap")
library("fBasics")
library("MuMIn")
library("MASS")
library("geiger")
library("geomorph")
library("vegan")
library("ggpubr")
library("rgl")
library("geometry")
library("webshot2")

#Load strict consensus tree
tree <- read.tree("abelis_SRCT.nex")
windows()
plot(tree)
#Read temporal data
ages <- read.table("FADLADalltax.txt", row.names = 1, sep = "\t", header = TRUE)

#Adjust taxon lists
equals <- intersect(rownames(ages),tree$tip.label)
temp_data.red <- ages[equals,]

#Generate temporal slices using stages
time.bins.intervals <- read.table("time_intervals.txt", row.names = 1, sep = "\t",
header = TRUE)

#Generate list of temporal ranges and matrix of taxa-bins
ranges_matrices <- binTimeData(temp_data.red,int.times=time.bins.intervals)

#Generate different matrices through subsampling
seqLists <- seqTimeList(ranges_matrices,nruns=100)

#Calculate sampling rate
sampPars <- t(sapply(seqLists$timeLists,function(x) optimPaleo(make_durationFreqDisc(x
))$par))
meanIntLen <- sapply(seqLists$timeLists,function(x) mean(-apply(x[[1]],1,diff)))
sampRate <- sapply(1:length(meanIntLen),function(x) sProb2sRate(R=sampPars[x,2],
int.length=meanIntLen[x]))
sampRate

#Delete infinite rates
sRateClean <- sampRate[!is.infinite(sampRate)]
nTaxaClean <- seqLists$nTaxa[!is.infinite(sampRate)]
nIntClean <- seqLists$nIntervals[!is.infinite(sampRate)]
meanIntLenClean <- meanIntLen[!is.infinite(sampRate)]
windows()
boxplot(sRateClean,ylab="Est. Sampling Rate per Lmy (Inf Results Dropped)")
mtext(text=paste("Median:",round(median(sRateClean),3)),1,2)

#Compare adjustment of different distributions
windows()
barplot(as.vector(Weights(c(
  IC(fitdistr(sRateClean,"gamma")$loglik,2,n=length(sRateClean)),
  IC(fitdistr(sRateClean,"exponential")$loglik,1,n=length(sRateClean)),
  IC(fitdistr(sRateClean,"log-normal")$loglik,2,n=length(sRateClean))
))),names=c("gamma","exponential","log-normal"),ylab="Akaike Weights")

#Log-normal distribution shows the best fit
modelFit.samp <- fitdistr(sRateClean,"log-normal")$estimate
```

```
#Calculate extinction rates
extRate <- sampPars[,1]/meanIntLen
extClean <- extRate[!is.infinite(sampRate)]
median(extClean)

#Compare adjustment of different distributions
windows()
barplot(as.vector(Weights(c(
  IC(fitdistr(extClean,"gamma")$loglik,2,n=length(extClean)),
  IC(fitdistr(extClean,"exponential")$loglik,1,n=length(extClean)),
  IC(fitdistr(extClean,"log-normal")$loglik,2,n=length(extClean))
))),names=c("gamma","exponential","log-normal"),ylab="Akaike Weights")

#Gamma distribution shows the best fit
modelFit.ext <- fitdistr(extClean,"gamma")$estimate

#Simulate and plot both rates
windows()
layout(1:2)
plot(sRateClean,extClean,xlab="Sampling Rate",ylab="Extinction Rate")
sampRateSim <- rexp(10,rate=modelFit.samp)
extRateSim <- rexp(10,rate=modelFit.ext)
windows()
plot(sampRateSim,extRateSim,xlab="Simulated Sampling Rate",ylab="Simulated Extinction Rate")

#Proceed with calibration
windows()
time.trees_cal3 <- list()
for (j in 1:length(sRateClean))  {
  time.trees_cal3[[j]] <- cal3TimePaleoPhy(tree, temp_data.red,
                                           ntrees = 10,
                                           anc.wt = 0,
                                           brRate = extClean[j],
                                           extRate = extClean[j],
                                           sampRate = sRateClean[j],
                                           plot = T)
}

#Combine all trees into a single list
time.trees_cal3_set <- list()
for (i in 1:length(sRateClean)) {
  time.trees_cal3_set <- c(time.trees_cal3_set, time.trees_cal3[[i]])
}

#Plot one of the trees as example
windows()
geoscalePhylo(ladderize(time.trees_cal3_set[[2]], right = T), ages = temp_data.red, x.lim = c(0, 300), cex.tip = 0.9, cex.age = 0.5, cex.ts = 0.6,
              label.offset = -1)

#Generate tree with mean ages
consesus.tree.cal3 <- consensus.edges(time.trees_cal3_set)
root.ages <- vector()
for (j in 1:length(time.trees_cal3_set)){
  root.ages[j] <- time.trees_cal3_set[[j]]$root.time
}
mean.root.ages <- mean(root.ages)

consesus.tree.cal3$root.time <- mean.root.ages
windows()
geoscalePhylo(ladderize(consesus.tree.cal3, right = T), ages = temp_data.red, x.lim = c(30, 250), cex.tip = 0.9, cex.age = 0.5, cex.ts = 0.6,
              label.offset = -1)

consesus.tree.cal3.red <- drop.tip(consesus.tree.cal3,tip = c("Indosaurus","Afromimus"))

#Transform any zero-length branch to 0.1
consesus.tree.cal3.red$edge.length[consesus.tree.cal3.red$edge.length == 0] <- 0.01
```

```
windows()
geoscalePhylo(ladderize(consesus.tree.cal3.red, right = T), ages = temp_data.red,
x.lim = c(30, 250), cex.tip = 0.9, cex.age = 0.5, cex.ts = 0.6,
              label.offset = -1)

write.tree(consesus.tree.cal3.red)
```
