# Supplementary material for: Morphology of the maxilla informs about the type of predation strategy in the evolution of Abelisauridae (Dinosauria: Theropoda)
Source: Sci Rep. 2025 Mar 6;15:7857. doi: 10.1038/s41598-025-87289-w (PMC11885552; doi:10.1038/s41598-025-87289-w)
Supplement: Supplementary file 9 — Supplementary Material 9 [file 41598_2025_87289_MOESM9_ESM.pdf]

	start_time	end_time
Late_Triassic	237	201.3
Early_Jurassic	201.3	174.1
Early_Jurassic1	201.3	190.8
Early_Jurassic2	199.3	182.7
Early_Jurassic3	190.8	174.1
Middle_Jurassic	174.1	163.5
Middle_Jurassic1	174.1	168.3
Middle_Jurassic2	168.3	163.5
Late_Jurassic	163.5	145
Late_Jurassic1	163.5	157.3
Late_Jurassic2	157.3	145
Early_Cretaceous	145	100.5
Early_Cretaceous1	145	125
Early_Cretaceous2	125	100.5
Late_Cretaceous	100.5	66
Cenomanian_Coniacian	100.5	86.3
Cenomanian_Turonian	100.5	89.8
Coniacian_Santonian	89.8	83.6
Santonian_Maastrichtian	86.3	66
Campanian_Maastrichtian	83.6	66
Maastrichtian	72.1	66
