# Supplementary material for: Morphology of the maxilla informs about the type of predation strategy in the evolution of Abelisauridae (Dinosauria: Theropoda)
Source: Sci Rep. 2025 Mar 6;15:7857. doi: 10.1038/s41598-025-87289-w (PMC11885552; doi:10.1038/s41598-025-87289-w)
Supplement: Supplementary file 10 — Supplementary Material 10 [file 41598_2025_87289_MOESM10_ESM.pdf]

```
macro-;
mxram 1200;
macro=;
taxname=;

warn-;
collapse3;

loop %2 %3

piwe=#1;

proc %1.tnt;

hold10000;

log+ output/%1_tree_searches_IW_k#1..out;

mu1000;bb;

log/;

tsave*output/%1_MPTs_IW_k#1..tre;
save;
tsave/;

randtrees/;
k1000;

tsave*output/%1_1K_MPTs_IW_k#1..tre;
save;
tsave/;

k1;
log output/%1_stats_IW_k#1..out;
fit;
length;
run STATSb.RUN;
log/;

stop

k0;

proc %1.tnt;

loop %2 %3

proc output/%1_1K_MPTs_IW_k#1..tre;

stop

ne*;
tchoose/;
ttags=;
tplot;
ttags & output/SCT_of_MPTs_of_all_IW_k.svg thick 3 italics legup -2 txtsep 16 ;
ttags-;
ttags);

quote =======================SCRIPT FINISHED SUCCESSFULLY=======================;

proc/;
```
