# Supplementary material for: Morphology of the maxilla informs about the type of predation strategy in the evolution of Abelisauridae (Dinosauria: Theropoda)
Source: Sci Rep. 2025 Mar 6;15:7857. doi: 10.1038/s41598-025-87289-w (PMC11885552; doi:10.1038/s41598-025-87289-w)
Supplement: Supplementary file 5 — Supplementary Material 5 [file 41598_2025_87289_MOESM5_ESM.pdf]

(((((((((((((((((((((Niebla:18.08012821,AbeliLaColonia:18.08012821)1:0.5951282051,Carnotaurus:18.67525641)1:1.369230769,Aucasaurus:15.44448718)1:2.899230769,Viavenator:5.643717949)1:2.747051282,Llukalkan:8.390769231)1:8.374615385,Skorpiovenator:10.26538462)1:0.4632051282,(Ekrixinatosaurus:6.598589744,Ilokelesia:10.29858974)1:0.43)1:0.8933333333,Arcovenator:30.82192308)1:1.782564103,MPM99:16.90448718)1:2.618846154,((Majungasaurus:11.58564103,Rajasaurus:12.46102564)1:9.87,Abelisaurus:20.72692308)1:14.49641026)1:5.869871795,Rugops:18.19320513)1:25.2624359,Spectrovenator:23.95564103)1:42.87961538,(Eoabelisaurus:1.706538462,Xenotarsosaurus:93.26653846)1:0.2687179487)1:0.6598717949,(((((CCG20011:7.281666667,Elaphrosaurus:69.08166667)1:1.576025641,Limusaurus:8.857692308)1:0.6167948718,MNNtig6:32.17448718)1:1.345,(Masiakasaurus:43.19217949,Noasaurus:43.46128205)0.526923076923077:54.89320513)1:3.267948718,Laevisuchus:103.3874359)1:11.10769231)1:1.388461538,Huinculsaurus:92.08358974)1:2.912307692,(Ceratosaurus:13.20307692,Genyodectes:63.50307692)1:20.79282051)1:9.147051282,Berberosaurus:19.84294872)1:3.098846154,Allosaurus:46.24179487)1:9.383717949,Dilophosaurus:23.72551282)1:8.137948718,Syntarsus:31.86346154)1:38.79294872,Herrerasaurus:26.35641026)1;
